# A head-to-head comparison of the EQ-5D-5L and 15D descriptive systems and index values in a general population sample

**DOI:** 10.1186/s12955-023-02096-z

**Published:** 2023-02-19

**Authors:** Anna Nikl, Mathieu F. Janssen, Valentin Brodszky, Fanni Rencz

**Affiliations:** 1 Department of Health Policy, Corvinus University of Budapest, 8 Fővám tér, Budapest, 1093 Hungary; 2 Károly Rácz Doctoral School of Clinical Medicine, Semmelweis University, Budapest, Hungary; 3 Section Medical Psychology and Psychotherapy, Department of Psychiatry, Erasmus MC, Rotterdam, The Netherlands

**Keywords:** Health-related quality of life, Health utility, EQ-5D-5L, 15D, Psychometrics

## Abstract

**Background:**

The EQ-5D-5L and 15D are generic preference-accompanied health status measures with similar dimensions. In this study, we aim to compare the measurement properties of the EQ-5D-5L and 15D descriptive systems and index values in a general population sample.

**Methods:**

In August 2021, an online cross-sectional survey was conducted in a representative adult general population sample (n = 1887). The EQ-5D-5L and 15D descriptive systems and index values were compared in terms of ceiling and floor, informativity (Shannon’s Evenness index), agreement, convergent and known-groups validity for 41 chronic physical and mental health conditions. Danish value sets were used to compute index values for both instruments. As a sensitivity analysis, index values were also estimated using the Hungarian EQ-5D-5L and Norwegian 15D value sets.

**Results:**

Overall, 270 (8.6%) and 1030 (3.4*10^−6^%) unique profiles occurred on the EQ-5D-5L and 15D. The EQ-5D-5L dimensions (0.51–0.70) demonstrated better informativity than those of 15D (0.44–0.69). EQ-5D-5L and 15D dimensions capturing similar areas of health showed moderate or strong correlations (0.558–0.690). The vision, hearing, eating, speech, excretion and mental function 15D dimensions demonstrated very weak or weak correlations with all EQ-5D-5L dimensions, which may indicate potential room for EQ-5D-5L bolt-ons. The 15D index values showed lower ceiling than the EQ-5D-5L (21% vs. 36%). The mean index values were 0.86 for the Danish EQ-5D-5L, 0.87 for the Hungarian EQ-5D-5L, 0.91 for the Danish 15D and 0.81 for the Norwegian 15D. Strong correlations were found between the index values (Danish EQ-5D-5L vs. Danish 15D 0.671, Hungarian EQ-5D-5L vs. Norwegian 15D 0.638). Both instruments were able to discriminate between all chronic condition groups with moderate or large effect sizes (Danish EQ-5D-5L 0.688–3.810, Hungarian EQ-5D-5L 1.233–4.360, Danish 15D 0.623–3.018 and Norwegian 15D 1.064–3.816). Compared to the 15D, effect sizes were larger for the EQ-5D-5L in 88–93% of chronic condition groups.

**Conclusions:**

This is the first study to compare the measurement properties of the EQ-5D-5L and 15D in a general population sample. Despite having 10 fewer dimensions, the EQ-5D-5L performed better than the 15D in many aspects. Our findings help to understand the differences between generic preference-accompanied measures and support resource allocation decisions.

**Supplementary Information:**

The online version contains supplementary material available at 10.1186/s12955-023-02096-z.

## Background

Generic preference-accompanied measures (PAMs) are health status measures that consist of two parts: the first is a descriptive system, and the second is a value set that allows assigning utilities to health profiles defined by the descriptive system. Over the past decades, an increasing number of generic PAMs have been developed, such as the EQ-5D, Short-Form 6-Dimension (SF-6D), Assessment of Quality of Life (AQoL) and Health Utilities Index (HUI) [1]. Despite the abundance of PAMs, the most commonly used one on an international level is the EQ-5D [2, 3]. In over 20 countries, national health technology assessment guidelines recommend the use of this instrument in cost-utility analyses of health interventions [4]. It was developed in the late 1980s by an international organization, the EuroQol Group, and currently, it has two versions for adults, the EQ-5D-3L and the newer EQ-5D-5L [5, 6]. Both versions showed good validity in several countries, languages, and patient populations [7, 8].

The 15D is a 15-dimensional generic PAM, which was developed in Finland starting from the 1970s [9]. The instrument has been validated in numerous patient populations and translated to multiple languages; however, its popularity is predominantly concentrated in the Nordic countries [10]. Country-specific 15D value sets have been developed in Finland [11], Denmark [12], and Norway [13, 14]. Two countries (Norway and Chile) mention the 15D as an acceptable instrument in their health technology assessment guidelines [15, 16]. It has also been used in several cost-effectiveness and cost-utility analyses in different countries and as part of health technology assessment dossiers, in conditions such as hip and knee arthrosis [17], Parkinson’s disease [18], cataract [19], acute and chronic liver failure [20, 21] and anorexia nervosa [22] in Finland, multiple myeloma in three Nordic countries (Denmark, Norway, Sweden) [23], stress urinary incontinence in Canada [24], and breast cancer in Iran [25].

Compared to the EQ-5D, the descriptive system of the 15D is considerably longer, more detailed, and comprehensive. Notwithstanding, the 15D and EQ-5D-5L instruments are similar in many aspects, which offers a strong basis for comparison. Firstly, a range of corresponding dimensions can be found between the two measures with similar wording, such as mobility, usual activities, pain/discomfort, and anxiety/depression/distress. Secondly, on both instruments each dimension of health has one item with five response levels measured on a severity or capability scale. Finally, both instruments investigate the current health status of the respondent. A few studies compared the measurement properties of the EQ-5D-3L and 15D in different patient populations, such as epilepsy [26], HIV/AIDS [27] and stroke [28] in Norway, multiple chronic conditions [29], chronic pain [30], critical care setting [31] and patients after cardiac surgery [32] in Finland. However, to date, only one study has examined the psychometric properties of the EQ-5D-5L and 15D, in a sample of Parkinson’s disease in Spain [33]. Furthermore, no studies have provided a comparison of the measurement properties of either the EQ-5D-3L or EQ-5D-5L and 15D in a general population sample.

Comparing PAMs in different populations is important to inform researchers, analysts and health policy decision-makers about their performance and support the choice of instrument for cost-utility analysis. Although the EQ-5D-5L has proved to be a valid instrument in a multitude of health conditions, it might not capture all important aspects of health, especially in sensory disorders [34] and mental health conditions [35]. Furthermore, a clear need emerged to include extra dimensions in the EQ-5D-5L, so-called “bolt-ons” [36]. In that respect, the 15D with its broader descriptive system may offer advantages over the EQ-5D-5L. On the other hand, the 15D needs to fit many more dimensions into the utility space, allowing on average less space for each separate dimension. Given the abovementioned similarities between the EQ-5D-5L and 15D, one may almost consider the 15D a variant of the EQ-5D-5L expanded with bolt-ons. Interestingly, five of the 15 dimensions of 15D have a corresponding EQ-5D dimension and further eight have earlier been proposed as possible bolt-ons for the EQ-5D (vision, sleeping, hearing, vitality, speech, breathing, mental function, and sexual activities) [37]. A comparative analysis between the two instruments may also provide new evidence that can later support the development of candidate bolt-on dimensions.

Therefore, in this study, we aim to conduct an exploratory analysis that compares the measurement properties of the EQ-5D-5L and 15D in a large general population sample in Hungary. We compare measurement properties of both the descriptive systems and the index values focusing on ceiling and floor effects, informativity, agreement, redistribution properties, convergent and known-groups validity.

## Methods

### Study design

A cross-sectional survey was conducted with a targeted sample size of 2000 members of the Hungarian adult general population (response rate 77.8%). The broader aim of the survey was to assess the mental health of the population. Permission for conducting the study was granted by the Research Ethics Committee of the Corvinus University of Budapest (no. KRH/166/2021). Participants were recruited in August 2021 from one of the largest available online panels in Hungary by a third-party survey company. Respondents registered voluntarily to complete surveys in return for points, which could be redeemed for rewards. Respondents were included who were at least 18 years old at the time of completion, gave informed consent, and confirmed that they had understood the terms and were willing to participate. ‘Soft’ quotas were applied to ensure the representativeness of the sample for the general population by age, gender, the highest level of education, geographical region, and settlement type.

### Outcome measures

A self-administered survey was designed for the study that asked questions about health-related quality of life, well-being, presence of physical and mental health conditions, resource utilization related to mental health care, and sociodemographic characteristics. The list of the physical health conditions was selected according to the 2019 Hungarian results of the European Health Interview Survey (EHIS) [38] complemented by some common chronic diseases. Similarly, the list of mental health conditions was chosen according to the fifth edition of the Diagnostic and Statistical Manual of Mental Disorders (DSM-5) [39]. We asked respondents to report any physical and mental health conditions experienced in the past 12 months in two questions. Firstly, they had to state whether they had any self-reported physical or mental health conditions. Secondly, they had to mark those that were also diagnosed by a physician. All participants completed a set of standardized questionnaires, including the validated Hungarian versions of EQ-5D-5L and 15D. The participants answered the questions in a fixed order, starting with the EQ-5D-5L and multiple questions were included between the EQ-5D-5L and 15D. As a base case, we used the Danish value sets for both the EQ-5D-5L [40] and 15D [12], because currently, Denmark is the only country with national value sets for both measures. However, using these value sets may have limitations. They were developed in different decades, using different preference elicitation methods, and thus have largely different value set ranges. Furthermore, using Danish value sets for Hungary may also pose additional problems given the differences in sociodemographic and economic characteristics and cultural values between the two countries [41]. Therefore, to test the robustness of our results, we repeated all analyses using the Hungarian EQ-5D-5L [42] and Norwegian 15D value sets [14]. The former was selected because of the study country, while the latter was considered as the most recently developed 15D value set with a similar value set range to the Hungarian EQ-5D-5L value set.

#### EQ-5D-5L

The EQ-5D-5L is a generic PAM that consists of two parts: a five-item descriptive system and a 0–100 visual analogue scale (EQ VAS) [5, 6]. The descriptive system contains five dimensions of health: mobility, self-care, usual activities, pain/discomfort and anxiety/depression, each with five response levels (no problems = 1, slight problems = 2, moderate problems = 3, severe problems = 4 and unable to/extreme problems = 5), allowing 5^5^ = 3125 unique health states in total [6]. Respondents are asked to recall their current health state (i.e., ‘your health today’). The Danish value set used as a base case in this study is based on a heteroscedastic censored hybrid model using both composite time trade-off (cTTO) and discrete choice experiment (DCE) data from a representative adult general population sample in Denmark (data collection period 2018–19) [40]. The lowest possible value with this value set is − 0.758, where negative values describe health states considered to be worse than dead and 1 refers to full health. The Hungarian value set that was used for the sensitivity analysis had been estimated by a pooled heteroscedastic Tobit model using cTTO data from a representative sample of the Hungarian adult general population (data collection period 2018–19) [42]. Index values range from − 0.848 to 1 with this value set.

#### 15D

The 15D is another generic PAM that covers 15 dimensions of health-related quality of life: mobility, vision, hearing, breathing, sleeping, eating, speech, excretion, usual activities, mental function, discomfort and symptoms, depression, distress, vitality, and sexual activities [9]. Each of these dimensions has five response levels (1 being the best possible health state of an individual, while 5 being the worst) with 5^15^ (more than 30 billion) possible distinct health states. The 15D asks respondents to recall their current health (i.e., ‘present health status’). The Danish value set was selected in this study as a base case. This was developed using an additive model of the multi-attribute utility theory based on valuations on a 0–100 visual analogue scale (VAS). Firstly, respondents were asked to weigh the top and bottom levels of each dimension individually on a VAS, then they were asked to assign a score to each level of each dimension on VAS (‘within dimension tasks’). Data were collected in 2001 and preferences of the non-institutionalized general population of Denmark aged 18–75 were assessed [12]. The index values of the final value set range from 0.160 to 1. The Norwegian value set, used for the sensitivity analysis, also relies on an additive model [14]. However, it only kept the ‘within dimension tasks’ from the original valuation that was supplemented by a pits-task, whereby respondents were asked to rate the worst possible health state on a VAS together with ‘being dead’. Data were collected in 2010 and 2015–16 from a representative sample of the Norwegian general population. The index values range from − 0.516 to 1.

### Statistical analyses

Our analytical framework builds on previous studies that compared the measurement properties of other generic PAMs [43–46]. As a result of a technical problem in the online survey interface, a few respondents’ EQ-5D-5L responses may have been inadvertently recorded as level 5 responses. Therefore, the research team examined all level 5 responses attentively in the EQ-5D-5L and compared them with other information (i.e. self-reported health status on other measures, physician-diagnosed physical and mental health conditions) provided by the respondents. As a result, 113 participants were excluded from the sample before the statistical analysis. To compare the two instruments, corresponding dimensions of EQ-5D-5L and 15D were matched, e.g. EQ-5D-5L mobility and 15D mobility. All analyses were performed on the total sample, and also for two subsets of respondents: (1) respondents with physical health conditions, and (2) respondents with mental health conditions. Statistical analyses were performed using R Statistical Software (version 4.1.1; R Foundation for Statistical Computing, Vienna, Austria). All the statistical tests were two-sided, and *p* < 0.05 was considered statistically significant.

#### Ceiling and floor

The proportion of participants reporting ‘no problems’ (ceiling) and ‘extreme problems’ (floor) was computed for each dimension of the descriptive systems. In addition, we calculated the ceiling and floor for the EQ-5D-5L and 15D health profiles, i.e. ‘no problems’ and ‘extreme problems’ in all dimensions, respectively. We expected a higher overall ceiling in the EQ-5D-5L than the 15D at an instrument level since the descriptive system of the latter is more detailed [28].

#### Informativity

The informativity of EQ-5D-5L and 15D dimensions, index values, and health state profiles was examined by Shannon’s (absolute informativity, H′) and Shannon’s Evenness (relative informativity, J′) indices [47, 48]. The Shannon index (H′) can be defined as

$$H^{\prime} = - \mathop \sum \limits_{i = 1}^{L} p_{i} *\log_{2} p_{i}$$

where *p*_i_ is the proportion of observations in the *i*th level (where i = 1, …, L), and L is the number of levels in a dimension of the descriptive system. The greatest amount of information can be gathered if the responses are equally used across the levels. The Shannon Evenness index (J′) measures the evenness of distribution and was calculated as

$$J^{\prime} = \frac{H^{\prime}}{{H^{\prime}_{max} }} = \frac{{ - \mathop \sum \nolimits_{i = 1}^{L} p_{i} *\log_{2} p_{i} }}{{\log_{2} L}}$$

Thus, H′ ranges from 0 to log_2_L, and J′ ranges from 0 to 1, where a higher value indicates better informativity.

#### Inconsistencies and agreement

We performed cross-tabulations of the corresponding EQ-5D-5L and 15D dimensions to explore how consistent the responses were. We considered an EQ-5D-5L and 15D response pair inconsistent if the 15D response was at least two levels away from the EQ-5D-5L response [49]. The average size of inconsistencies was assessed according to the following weights: 0 if EQ-5D-5L and 15D responses did not differ more than 1 level, 1 if responses differed by 2 levels, and so forth [49].

The agreement between the EQ-5D-5L and 15D index values was examined using intraclass correlation coefficient (ICC) [50] and Bland–Altman plot [51]. A two-way random model with absolute agreement was applied to obtain an ICC value [52]. Agreement was considered poor 0 ≤ ICC < 0.4, fair 0.4 ≤ ICC < 0.6, good 0.6 ≤ ICC < 0.75, and excellent 0.75 ≤ ICC < 1 [53].

#### Convergent validity

We examined the convergent validity between the EQ-5D-5L and 15D dimensions (Spearman’s correlation) and index values (Pearson’s correlation). The absolute value of the correlation coefficient (r) was interpreted as follows: very weak correlation |r| < 0.2, weak correlation 0.2 ≤ |r| < 0.4, moderate correlation 0.4 ≤ |r| < 0.6 and strong correlation 0.6 ≤ |r| ≤ 1 [54]. We expected higher correlations among the corresponding dimensions covering similar aspects of health [26].

#### Known-groups validity

Known-groups validity was evaluated for self-reported physician-diagnosed health condition groups in contrast to being healthy. We hypothesized that respondents with a diagnosed physical or mental condition had significantly lower EQ-5D-5L and 15D index values. Student’s *t* test was used to compare the healthy and non-healthy groups. Effect size (ES, Cohen’s d) and relative efficiency (RE) were calculated. ES values were interpreted as negligible d < 0.2, small 0.2 ≤ d < 0.5, medium 0.5 ≤ d < 0.8, and large 0.8 ≤ d [55]. The RE was calculated as the ESs ratio of the two indices, where the 15D test statistic was used as reference; thus, a RE > 1 indicated that the EQ-5D-5L was more efficient in discriminating between two subgroups. To test whether the RE statistically differs from 1, 95% confidence intervals were calculated using 2000 bootstrap samples with accelerated bias correction.

## Results

### Characteristics of the study population

The distribution of the sample (n = 1887) reasonably approximated that of the general population in terms of sociodemographics (Additional file 1: Supplementary material 1). Altogether 63.4% of the sample responded that they had one or more physical conditions and 35.2% reported at least one mental health condition diagnosed by a physician.

### Dimension-level analysis

As for the EQ-5D-5L dimensions, the floor varied between 0.2% (usual activities) and 1.2% (anxiety/depression), while the ceiling ranged from 50.8% (pain/discomfort) to 87.7% (self-care) (Table 1). Regarding the 15D dimensions, the floor reached its lowest at 0.2% (eating) and its highest at 3.9% (sexual activities), while for the ceiling, the values varied between 48.4% (sleeping) and 94.4% (eating). The EQ-5D-5L had lower ceiling in all corresponding dimension pairs, except for the EQ-5D-5L anxiety/depression vs. 15D distress pair. The highest difference in ceiling was found between EQ-5D-5L pain/discomfort (50.8%) and 15D discomfort and symptoms (68.2%). Similarly, the floor was equal or lower in the EQ-5D-5L for all pairs but EQ-5D-5L anxiety/depression vs. 15D depression. The largest difference in floor was seen between EQ-5D-5L anxiety/depression (1.2%) and 15D distress (1.7%).

**Table 1:** Floor and ceiling of EQ-5D-5L and 15D

EQ-5D-5L							15D						
Dimensions	Total sample (N = 1887)		Physical conditions (N = 1195)		Mental conditions (N = 664)		Dimensions	Total sample (N = 1887)		Physical conditions (N = 1195)		Mental conditions (N = 664)	
	Ceiling n (%)	Floor n (%)	Ceiling n (%)	Floor n (%)	Ceiling n (%)	Floor n (%)		Ceiling n (%)	Floor n (%)	Ceiling n (%)	Floor n (%)	Ceiling n (%)	Floor n (%)
Mobility (walking)	1246 (66.0)	7 (0.4)	670 (56.1)	5 (0.4)	359 (54.1)	3 (0.5)	Mobility (walking, moving about)	1054 (78.0)	14 (0.7)	877 (73.4)	6 (0.5)	467 (70.3)	2 (0.3)
Self-care (washing or dressing)	1654 (87.7)	9 (0.5)	1027 (85.9)	8 (0.7)	538 (81.0)	4 (0.6)	–	–	–	–	–	–	–
Usual activities (e.g. work, study, housework, family or leisure activities)	1393 (73.8)	4 (0.2)	798 (66.8)	2 (0.2)	415 (62.5)	2 (0.3)	Usual activities (e.g. employment, studying, housework, free-time activities)	1467 (77.7)	8 (0.4)	857 (71.7)	1 (0.1)	436 (65.7)	1 (0.2)
Pain/discomfort	959 (50.8)	9 (0.5)	474 (39.7)	7 (0.6)	226 (34.0)	8 (1.2)	Discomfort and symptoms (e.g. pain, ache, nausea, itching etc.)	1287 (68.2)	9 (0.5)	719 (60.2)	2 (0.2)	355 (53.5)	4 (0.6)
Anxiety/depression	1147 (60.8)	23 (1.2)	675 (56.5)	16 (1.3)	272 (41.0)	16 (2.4)	Depression (sad, melancholic or depressed)	1295 (68.6)	21 (1.1)	777 (65.0)	12 (1.0)	343 (51.7)	10 (1.5)
							Distress (anxious, stressed or nervous)	1054 (55.9)	33 (1.7)	607 (50.8)	19 (1.6)	262 (39.5)	18 (2.7)
–	–	–	–	–	–	–	Vision (seeing and reading with or without glasses)	1360 (72.1)	17 (0.9)	812 (67.9)	5 (0.4)	408 (61.4)	6 (0.9)
							Hearing (with or without a hearing aid)	1581 (83.8)	6 (0.3)	966 (80.8)	2 (0.2)	512 (77.1)	1 (0.2)
							Breathing (breathing difficulties, shortness of breath)	1342 (71.1)	21 (1.1)	765 (60.4)	17 (1.4)	379 (57.1)	13 (2.0)
							Sleeping	921 (48.4)	14 (0.7)	491 (41.1)	9 (0.8)	225 (33.9)	9 (1.4)
							Eating	1781 (94.4)	3 (0.2)	1150 (96.2)	0 (0.0)	608 (91.6)	0 (0.0)
							Speech	1701 (90.1)	5 (0.3)	1084 (90.7)	2 (0.2)	564 (84.9)	2 (0.3)
							Excretion (bladder and bowel)	1399 (74.1)	14 (0.7)	814 (68.1)	7 (0.6)	427 (64.3)	6 (0.9)
							Mental function (thinking clearly and logically, memory)	1596 (84.6)	7 (0.4)	989 (82.8)	1 (0.1)	504 (75.9)	2 (0.3)
							Vitality (e.g. healthy and energetic, weary, tired or feeble, exhausted)	950 (50.3)	20 (1.1)	502 (42.0)	10 (0.8)	240 (36.1)	12 (1.8)
							Sexual activities	1313 (69.6)	73 (3.9)	735 (61.5)	66 (5.5)	373 (56.2)	46 (6.9)
EQ-5D-5L index value^a^	679 (36.0)	0 (0.0)	305 (25.5)	0 (0.0)	124 (18.7)	0 (0.0)	15D index value^a^	396 (21.0)	1 (0.1)	147 (12.3)	0 (0.0)	67 (10.1)	0 (0.0)
EQ VAS	105 (5.6)	3 (0.2)	31 (2.6)	2 (0.2)	19 (2.9)	2 (0.3)	–	–	–	–	–	–	–

^a^Note that ceiling and floor are identical regardless of the value set used

EQ-5D-5L outperformed 15D regarding relative informativity (J′) for all dimensions (ranging from 0.51 to 0.70 for the EQ-5D-5L and from 0.44 to 0.69 for the 15D), except for the EQ-5D-5L anxiety/depression (0.65) vs. 15D distress (0.69) (Table 2). Considering all dimensions of each instrument, the average J′ values showed better results for the EQ-5D-5L (0.56) than for the 15D (0.49).

**Table 2:** Relative informativity of EQ-5D-5L and 15D (Shannon’s Evenness index)

EQ-5D-5L				15D			
Dimensions	Total sample (N = 1887)	Physical conditions (N = 1195)	Mental conditions (N = 664)	Dimensions	Total sample (N = 2000)	Physical conditions (N = 1195)	Mental conditions (N = 664)
Mobility (walking)	0.61	0.71	0.72	Mobility (walking, moving about)	0.44	0.49	0.52
Self-care (washing or dressing)	0.31	0.35	0.43	–	–	–	–
Usual activities (e.g. work, study, housework, family or leisure activities)	0.51	0.59	0.64	Usual activities (e.g. employment, studying, housework, free-time activities)	0.45	0.50	0.59
Pain/discomfort	0.70	0.76	0.81	Discomfort and symptoms (e.g. pain, ache, nausea, itching etc.)	0.55	0.61	0.69
Anxiety/depression	0.65	0.69	0.81	Depression (sad, melancholic or depressed)	0.57	0.60	0.73
				Distress (anxious, stressed or nervous)	0.69	0.71	0.82
–	–	–	–	Vision (seeing and reading with or without glasses)	0.52	0.55	0.63
				Hearing (with or without a hearing aid)	0.36	0.39	0.45
				Breathing (breathing difficulties, shortness of breath)	0.52	0.58	0.66
				Sleeping	0.70	0.74	0.82
				Eating	0.17	0.12	0.23
				Speech	0.25	0.23	0.35
				Excretion (bladder and bowel)	0.47	0.51	0.58
				Mental function (thinking clearly and logically, memory)	0.34	0.34	0.45
				Vitality (e.g. healthy and energetic, weary, tired or feeble, exhausted)	0.71	0.74	0.82
				Sexual activities	0.60	0.69	0.76
Total average	0.56	0.62	0.68	Total average	0.49	0.52	0.61

Responses covered all levels in both the EQ-5D-5L and 15D among the corresponding dimensions (Additional file 1: Supplementary materials 2–5). The rate of inconsistent response pairs was ranging from 4.6% (EQ-5D-5L anxiety/depression and 15D depression) to 7.9% (EQ-5D-5L mobility and 15D mobility). The average size of inconsistency was relatively low, ranging from 1.20 to 1.24.

As for the corresponding dimensions, we observed strong correlation between the EQ-5D-5L and 15D usual activities dimensions (0.619) (Table 3). The EQ-5D-5L anxiety/depression correlated stronger with 15D depression (0.690) than with 15D distress (0.642). Moderate correlation was found between the two mobility dimensions (0.558), as well as between the EQ-5D-5L dimension pain/discomfort and the 15D dimension discomfort and symptoms (0.583). The non-corresponding dimension pairs were correlated weakly to moderately, ranging from 0.115 (EQ-5D-5L mobility and 15D eating) to 0.541 (EQ-5D-5L pain/discomfort and 15D vitality). We observed moderate correlation between the EQ VAS and all EQ-5D-5L domains (except for self-care, where correlation was weak), while mostly weak and moderate connection with the 15D dimensions.

**Table 3:** Correlation coefficients between 15D and EQ-5D-5L items

	EQ-5D-5L					EQ VAS	EQ-5D-5L index value (Danish)	15D index value (Danish)	EQ-5D-5L index value (Hungarian)	15D index value (Norwegian)
	Mobility	Self-care	Usual activities	Pain/discomfort	Anxiety/depression					
15D										
Mobility	**0.558**	0.459	0.534	0.405	0.220	− 0.401	− 0.456	− 0.549	− 0.490	− 0.535
Vision	0.295	0.271	0.310	0.310	0.260	− 0.317	− 0.352	− 0.511	− 0.354	− 0.513
Hearing	0.236	0.288	0.258	0.230	0.176	− 0.239	− 0.267	− 0.434	− 0.277	− 0.430
Breathing	0.388	0.301	0.412	0.372	0.293	− 0.354	− 0.415	− 0.627	− 0.424	− 0.615
Sleeping	0.280	0.209	0.311	0.446	0.431	− 0.351	− 0.480	− 0.668	− 0.464	− 0.673
Eating	0.115	0.300	0.179	0.122	0.165	− 0.136	− 0.176	− 0.346	− 0.174	− 0.342
Speech	0.154	0.285	0.230	0.191	0.277	− 0.187	− 0.267	− 0.425	− 0.256	− 0.420
Excretion	0.274	0.229	0.296	0.340	0.264	− 0.297	− 0.358	− 0.555	− 0.358	− 0.566
Usual activities	0.480	0.459	**0.619**	0.481	0.357	− 0.453	− 0.537	− 0.643	− 0.548	− 0.640
Mental function	0.240	0.293	0.299	0.322	0.372	− 0.266	− 0.383	− 0.535	− 0.370	− 0.528
Discomfort and symptoms	0.411	0.308	0.447	**0.583**	0.472	− 0.471	− 0.588	− 0.708	− 0.578	− 0.711
Depression	0.218	0.228	0.309	0.410	**0.690**	− 0.374	− 0.571	− 0.679	− 0.519	− 0.687
Distress	0.218	0.168	0.293	0.416	**0.642**	− 0.363	− 0.548	− 0.680	− 0.500	− 0.702
Vitality	0.380	0.275	0.460	0.541	0.492	− 0.510	− 0.596	− 0.782	− 0.581	− 0.785
Sexual activities	0.374	0.268	0.428	0.430	0.334	− 0.391	− 0.461	− 0.632	− 0.463	− 0.637
EQ VAS	− 0.471	− 0.327	− 0.474	− 0.572	− 0.411	–	–	–	–	–
EQ-5D-5L index value (Danish)	− 0.661	− 0.482	− 0.663	− 0.829	− 0.767	0.604	–	–	–	–
15D index value (Danish)	− 0.485	− 0.369	− 0.530	− 0.629	− 0.578	0.534	0.671	–	–	–
EQ-5D-5L index value (Hungarian)	− 0.710	− 0.516	− 0.695	− 0.845	− 0.681	0.604	0.963	0.639	–	–
15D index value (Norwegian)	− 0.479	− 0.361	− 0.524	− 0.629	− 0.586	0.542	0.671	0.998	0.638	–

Pearson’s correlation coefficient was calculated for the continuous index values, while Spearman’s rank correlation for the ordinal dimensions

*p* < 0.05 for all correlation coefficients (two-tailed)

Corresponding dimensions between EQ-5D-5L and 15D are in bold

### Analysis of the index values

**Fig. 1:**
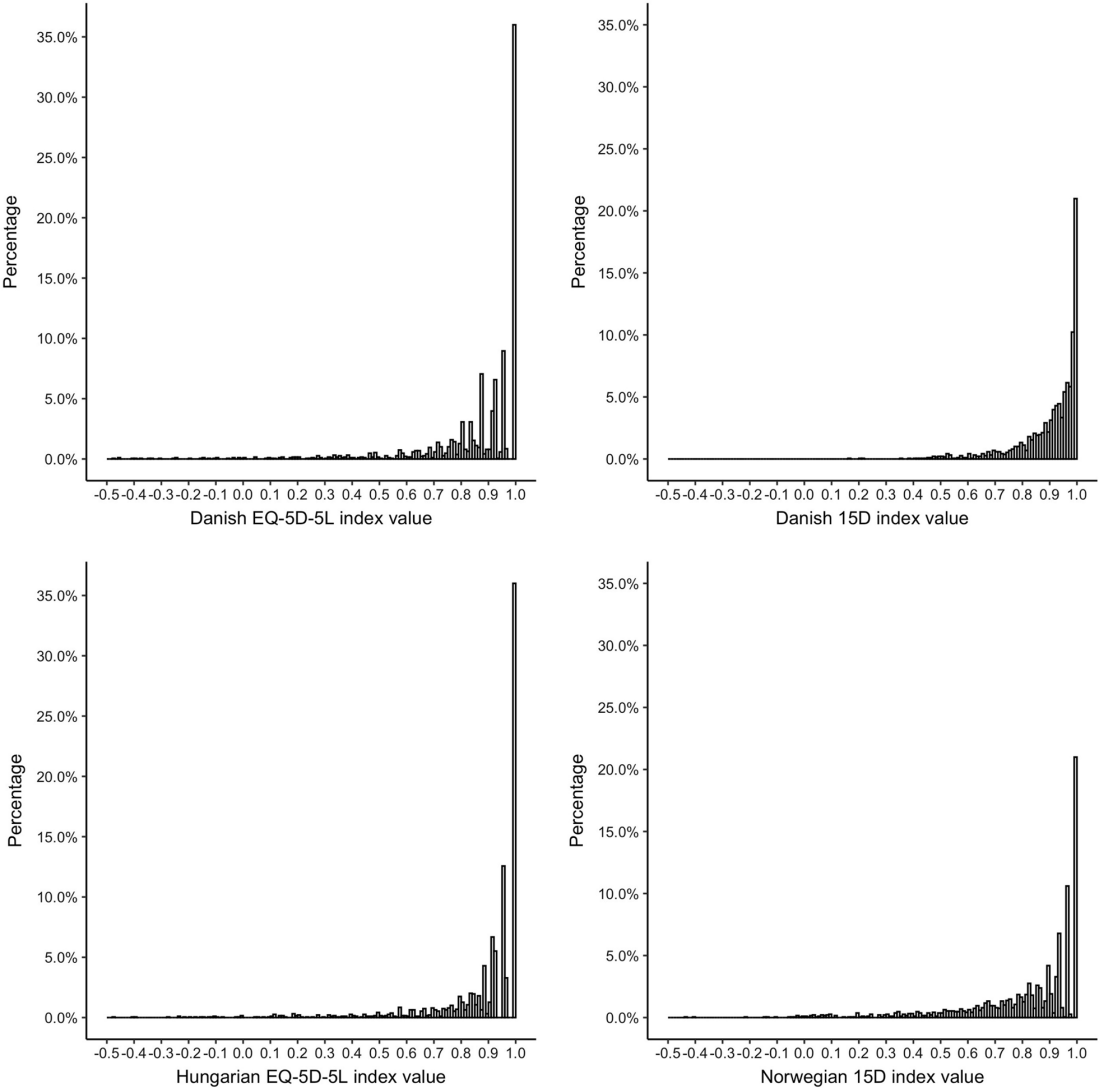
Distribution of EQ-5D-5L and 15D index values

The distributions of the EQ-5D-5L and 15D index values are presented in Fig. 1, while the main characteristics of the indices can be found in Table 4. Overall, 270 unique health states were observed for the EQ-5D-5L and 1030 for the 15D. The most common health state profile for both instruments was full health, accounting for 36.0% of the EQ-5D-5L answers and 21.0% of the 15D answers. As for the EQ-5D-5L, the second most common profile was slight pain or discomfort with no problems on the other dimensions (6.4%), while for the 15D, slight problems with sleeping and no other problems (3.2%).

**Table 4:**
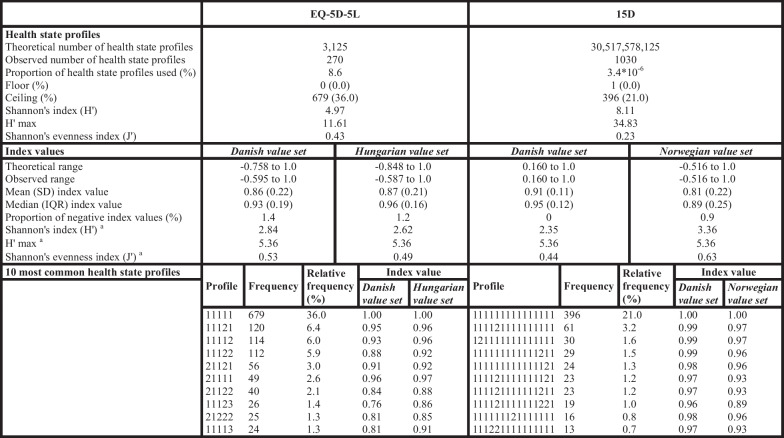
Characteristics of EQ-5D-5L and 15D health state profiles and index values

Order of domains for the EQ-5D-5L: mobility, self-care, usual activities, pain/discomfort, anxiety/depression

Order of domains for the 15D: mobility, vision, hearing, breathing, sleeping, eating, speech, excretion, usual activities, mental function, discomfort and symptoms, depression, distress, vitality, sexual activities

^a^To allow for comparisons between the two instruments, we split the utility scale with a bin width of 0.05 between − 1.0 and 1.0, resulting in a total of 41 intervals

In the total sample, the mean index value was the highest using the Danish 15D (0.91, SD = 0.11), followed by the Hungarian EQ-5D-5L (0.87, SD = 0.21), the Danish EQ-5D-5L (0.86, SD = 0.22), and the Norwegian 15D value set (0.81, SD = 0.22). The floor was negligible for 15D and not present for the EQ-5D-5L. For the Danish EQ-5D-5L, 1.4% of the index values were in the negative range, while for the Danish 15D, the theoretical minimum is higher than 0. However, 1.2% of the Hungarian EQ-5D-5L and 0.9% of the Norwegian 15D index values were negative. When the index value range was split with a bin width of 0.05, the Norwegian 15D showed the best relative informativity (J′) (0.63), followed by the Danish EQ-5D-5L (0.53), the Hungarian EQ-5D-5L (0.49), while the lowest J′ was demonstrated by the Danish 15D (0.44) (Table 4).

**Fig. 2:**
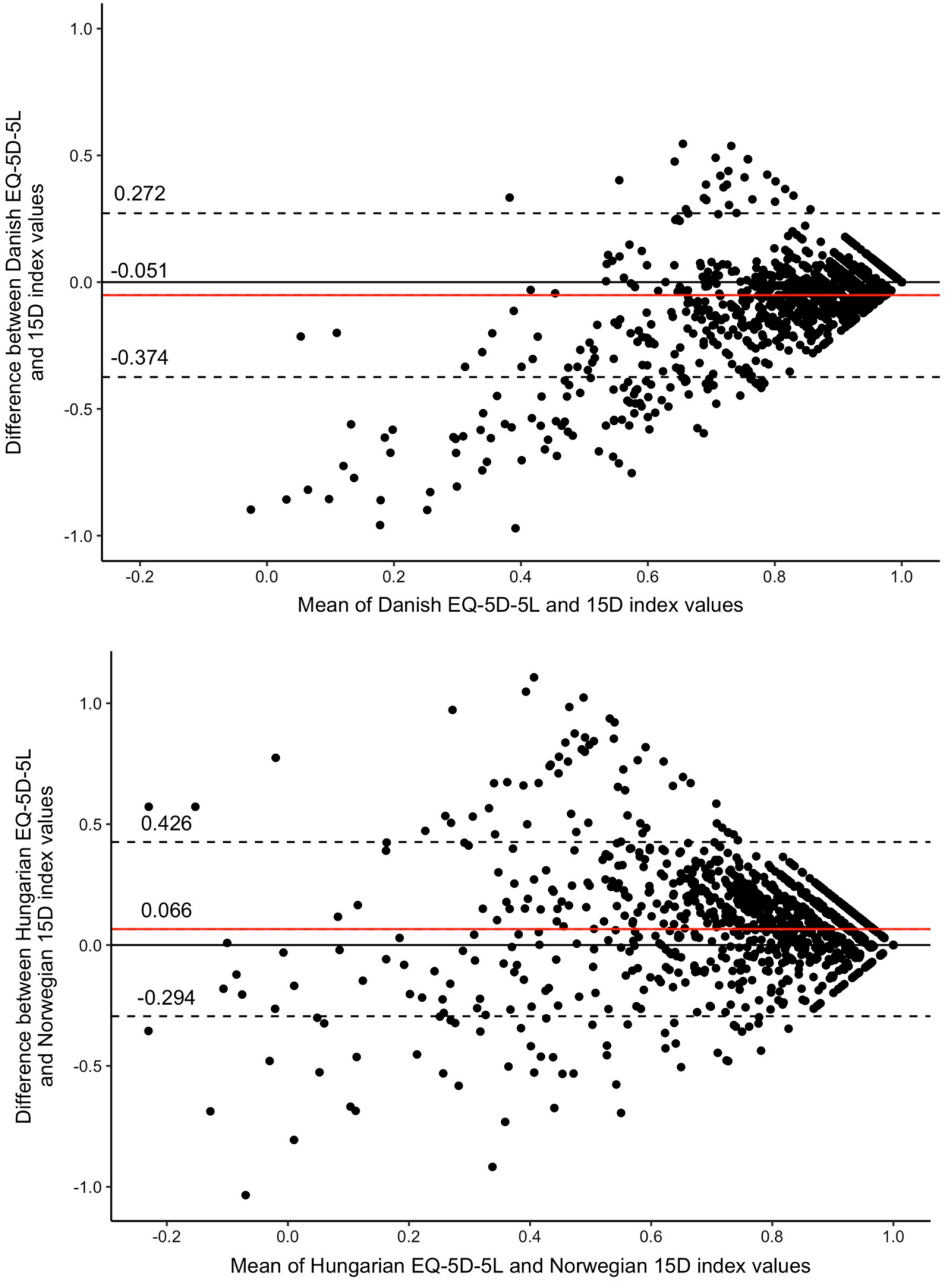
Bland–Altman plot of EQ-5D-5L and 15D index values. The horizontal red line represents the mean of the differences (D) between EQ-5D-5L and 15D index values, while the 95% confidence interval is represented by the dashed lines, which was obtained as D ± 1.96*SD (SD: standard deviation of the differences)

Poor agreement was found between the Danish EQ-5D-5L and 15D index values with an ICC of 0.363 (95% confidence interval: 0.342 to 0.385, *p* < 0.001) but a good agreement was found between the Hungarian EQ-5D-5L and Norwegian 15D index values with an ICC of 0.607 (95%CI 0.516–0.677, *p* < 0.001). The Bland–Altman plot indicated that 93.3% of the points lay within the 95% limits of agreement between the Danish EQ-5D-5L and 15D (94.2% between the Hungarian EQ-5D-5L and Norwegian 15D). Differences between the EQ-5D-5L and 15D index values increased at lower mean values for both value set pairs (Fig. 2).

Using the Danish value sets, a strong correlation was found between the EQ-5D-5L and 15D index values (0.671), and the EQ-5D-5L index value and EQ VAS value (0.604), while a moderate correlation was found between the 15D index value with the EQ VAS (0.534). The EQ-5D-5L index value demonstrated a strong correlation with its dimensions, except for self-care, where the correlation was moderate (− 0.482). By contrast, correlation coefficients between 15D dimensions and the EQ-5D-5L index value were ranging from − 0.596 (vitality) to − 0.176 (eating). 15D index value correlated moderately or strongly with most of its dimensions, while only weakly with the eating dimension (− 0.346). Considering the EQ-5D-5L dimensions with the 15D index value, the strongest correlation was observed for the pain/discomfort dimension (− 0.629), while the weakest for self-care (− 0.369). The convergent validity results were confirmed by the sensitivity analysis (Table 3).

Both the Danish EQ-5D-5L and 15D index values were able to discriminate between all chronic condition groups with moderate or large effect sizes (ranging from 0.688 to 3.810 for the EQ-5D-5L and from 0.623 to 3.018 for the 15D) (Table 5). Overall, the EQ-5D-5L was able to discriminate more effectively between 38/41 (93%) known-groups (RE > 1). Nevertheless, the bootstrap analysis suggested that results were significant in only five condition groups, dementia (RE = 1.465), other physical health conditions (RE = 1.448), bipolar depression (RE = 1.385), thyroid diseases (RE = 1.269), and gastroesophageal reflux disease (RE = 1.251). Using the Hungarian EQ-5D-5L and the Norwegian 15D value sets, effect sizes were large in all condition groups, and RE was > 1 in 36/41 (88%) known-groups. However, according to the results of the bootstrap analysis, the difference was only significant in four condition groups: dementia (RE = 1.672), chronic kidney disease (RE = 1.456), other physical health conditions (RE = 1.454), and urinary incontinence (RE = 1.302) (Table 6).

**Table 5:** Known-groups validity of the EQ-5D-5L and 15D (Danish value sets)

	n (%)	EQ-5D-5L				15D				RE^b^	95% CI^c^
		Mean (SD)	Median (Q1-Q3)	*p* value^a^	Cohen’s d ES	Mean (SD)	Median (Q1-Q3)	*p* value^a^	Cohen’s d ES		
Healthy	383 (20.3)	0.94 (0.12)	1.0 (0.93–1.00)	–	–	0.95 (0.10)	0.99 (0.95–1.0)	–	–	–	–
Physical conditions											
Hypertension	527 (27.9)	0.79 (0.27)	0.88 (0.75–0.96)	< 0.001	0.696	0.88 (0.11)	0.91 (0.83–0.97)	< 0.001	0.650	1.071	0.884–1.365
Musculoskeletal diseases	461 (24.4)	0.73 (0.29)	0.83 (0.67–0.91)	< 0.001	0.922	0.86 (0.11)	0.89 (0.80–0.95)	< 0.001	0.844	1.092	0.930–1.350
Allergies	318 (16.9)	0.82 (0.24)	0.89 (0.76–1.0)	< 0.001	0.697	0.89 (0.11)	0.92 (0.84–0.97)	< 0.001	0.623	1.119	0.893–1.517
Cardiovascular disease	259 (13.7)	0.70 (0.31)	0.81 (0.62–0.92)	< 0.001	1.134	0.83 (0.13)	0.85 (0.75–0.93)	< 0.001	1.082	1.048	0.893–1.260
Gastrointestinal or hepatic disease	241 (12.8)	0.74 (0.30)	0.84 (0.65–0.93)	< 0.001	0.993	0.85 (0.13)	0.88 (0.79–0.95)	< 0.001	0.894	1.111	0.924–1.389
Hyperlipidaemia	240 (12.7)	0.77 (0.28)	0.86 (0.72–0.95)	< 0.001	0.882	0.86 (0.12)	0.89 (0.80–0.96)	< 0.001	0.836	1.056	0.869–1.334
Eye or visual diseases	231 (12.2)	0.73 (0.29)	0.83 (0.64–0.93)	< 0.001	1.079	0.83 (0.13)	0.84 (0.76–0.93)	< 0.001	1.111	0.971	0.813–1.170
Diabetes	205 (10.9)	0.76 (0.31)	0.86 (0.72–0.96)	< 0.001	0.902	0.86 (0.13)	0.89 (0.80–0.97)	< 0.001	0.784	1.152	0.930–1.502
Gastroesophageal reflux disease	186 (9.9)	0.74 (0.30)	0.84 (0.67–0.93)	< 0.001	1.045	0.86 (0.12)	0.89 (0.80–0.96)	< 0.001	0.834	1.251	1.012–1.619
Respiratory diseases	175 (9.3)	0.79 (0.28)	0.88 (0.71–0.95)	< 0.001	0.861	0.85 (0.12)	0.88 (0.79–0.94)	< 0.001	0.905	0.952	0.763–1.227
Arrhythmias	172 (9.1)	0.71 (0.28)	0.8 (0.64–0.91)	< 0.001	1.284	0.82 (0.13)	0.84 (0.75–0.92)	< 0.001	1.154	1.112	0.913–1.389
Thyroid diseases	171 (9.1)	0.78 (0.27)	0.88 (0.72–0.95)	< 0.001	0.930	0.87 (0.12)	0.90 (0.84–0.96)	< 0.001	0.732	1.269	1.007–1.689
Skin diseases	166 (8.8)	0.78 (0.30)	0.88 (0.76–0.95)	< 0.001	0.869	0.86 (0.12)	0.90 (0.80–0.96)	< 0.001	0.809	1.074	0.867–1.402
Headache, migraine	139 (7.4)	0.71 (0.33)	0.81 (0.64–0.93)	< 0.001	1.175	0.84 (0.14)	0.88 (0.77–0.96)	< 0.001	0.987	1.190	0.961–1.499
Hearing impairment	133 (7.1)	0.73 (0.31)	0.84 (0.66–0.93)	< 0.001	1.153	0.84 (0.13)	0.89 (0.77–0.94)	< 0.001	0.982	1.174	0.959–1.515
Benign prostate hyperplasia	88 (4.7)	0.80 (0.26)	0.88 (0.77–0.95)	< 0.001	0.958	0.86 (0.11)	0.89 (0.81–0.95)	< 0.001	0.871	1.099	0.774–1.532
Urinary incontinence	71 (3.8)	0.68 (0.35)	0.79 (0.57–0.92)	< 0.001	1.538	0.81 (0.15)	0.85 (0.72–0.91)	< 0.001	1.282	1.199	0.967–1.525
Cancer, leukemia, lymphoma	46 (2.4)	0.73 (0.31)	0.85 (0.65–0.93)	< 0.001	1.416	0.83 (0.14)	0.87 (0.75–0.94)	< 0.001	1.098	1.290	0.951–1.828
Chronic kidney disease	29 (1.5)	0.71 (0.27)	0.83 (0.64–0.93)	< 0.001	1.735	0.83 (0.13)	0.85 (0.77–0.92)	< 0.001	1.209	1.435	0.986–2.022
Epilepsy	17 (0.9)	0.62 (0.39)	0.72 (0.54–0.93)	0.003	2.309	0.79 (0.16)	0.8 (0.70–0.92)	< 0.001	1.543	1.497	0.905–2.308
Liver cirrhosis	14 (0.7)	0.63 (0.44)	0.77 (0.43–0.99)	0.019	2.259	0.78 (0.19)	0.79 (0.67–0.95)	0.005	1.645	1.373	0.968–1.849
Other physical health conditions	92 (4.9)	0.76 (0.24)	0.84 (0.64–0.95)	< 0.001	1.236	0.86 (0.11)	0.89 (0.80–0.94)	< 0.001	0.854	1.448	1.075–2.008
Mental conditions											
Smoking addiction	381 (20.2)	0.80 (0.27)	0.88 (0.76–0.96)	< 0.001	0.688	0.88 (0.12)	0.92 (0.84–0.97)	< 0.001	0.628	1.096	0.896–1.442
Anxiety, phobia, or panic disorder	172 (9.1)	0.64 (0.32)	0.72 (0.51–0.88)	< 0.001	1.506	0.79 (0.15)	0.82 (0.69–0.90)	< 0.001	1.401	1.075	0.910–1.308
Sleeping disorders	169 (9.0)	0.65 (0.32)	0.76 (0.57–0.88)	< 0.001	1.459	0.81 (0.13)	0.84 (0.72–0.91)	< 0.001	1.253	1.164	0.969–1.440
Other addictions ^d^	98 (5.2)	0.70 (0.34)	0.81 (0.59–0.93)	< 0.001	1.342	0.82 (0.17)	0.86 (0.72–0.93)	< 0.001	1.155	1.162	0.938–1.474
Depression or dysthymia	79 (4.2)	0.54 (0.35)	0.68 (0.35–0.80)	< 0.001	2.228	0.75 (0.14)	0.78 (0.66–0.84)	< 0.001	1.861	1.198	0.953–1.496
Alcohol addiction	73 (3.9)	0.75 (0.29)	0.86 (0.67–0.93)	< 0.001	1.227	0.82 (0.16)	0.86 (0.74–0.93)	< 0.001	1.167	1.052	0.765–1.415
Substance addiction	55 (2.9)	0.63 (0.36)	0.75 (0.55–0.90)	< 0.001	1.870	0.77 (0.19)	0.81 (0.65–0.92)	< 0.001	1.533	1.220	0.955–1.595
Sexual disorder	40 (2.1)	0.71 (0.34)	0.82 (0.60–0.93)	< 0.001	1.545	0.79 (0.15)	0.82 (0.72–0.88)	< 0.001	1.524	1.014	0.729–1.349
Bipolar depression	35 (1.9)	0.60 (0.32)	0.68 (0.36–0.85)	< 0.001	2.398	0.76 (0.16)	0.81 (0.64–0.87)	< 0.001	1.732	1.385	1.019–1.859
Personality disorder	31 (1.6)	0.53 (0.35)	0.64 (0.35–0.78)	< 0.001	2.815	0.71 (0.16)	0.74 (0.63–0.82)	< 0.001	2.257	1.248	0.904–1.667
Learning disability	28 (1.5)	0.71 (0.35)	0.86 (0.64–0.97)	0.002	1.590	0.80 (0.21)	0.87 (0.66–0.96)	0.001	1.377	1.155	0.678–1.707
Eating disorder	26 (1.4)	0.64 (0.39)	0.78 (0.50–0.90)	< 0.001	2.060	0.78 (0.19)	0.86 (0.61–0.92)	< 0.001	1.572	1.310	0.934–1.810
Obsessive compulsive disorder	21 (1.1)	0.49 (0.43)	0.72 (0.33–0.78)	< 0.001	3.027	0.67 (0.17)	0.70 (0.53–0.83)	< 0.001	2.614	1.158	0.711–1.712
Dementia	18 (1.0)	0.44 (0.32)	0.47 (0.32–0.63)	< 0.001	3.810	0.68 (0.15)	0.66 (0.54–0.80)	< 0.001	2.601	1.465	1.035–2.085
Psychotic disorders	17 (0.9)	0.61 (0.42)	0.76 (0.43–0.92)	0.005	2.314	0.67 (0.21)	0.65 (0.51–0.84)	< 0.001	2.551	0.907	0.443–1.437
Post-traumatic stress disorder	14 (0.7)	0.49 (0.28)	0.51 (0.35–0.66)	< 0.001	3.569	0.64 (0.18)	0.66 (0.50–0.75)	< 0.001	2.955	1.208	0.787–1.751
Impulse-control disorder	14 (0.7)	0.56 (0.41)	0.64 (0.34–0.93)	0.004	2.772	0.70 (0.17)	0.65 (0.61–0.83)	< 0.001	2.356	1.177	0.666–1.813
Autism spectrum disorder	11 (0.6)	0.50 (0.36)	0.52 (0.38–0.79)	0.002	3.463	0.64 (0.22)	0.67 (0.46–0.80)	0.001	2.902	1.193	0.655–1.911
Attention deficit hyperactivity disorder	10 (0.5)	0.54 (0.36)	0.66 (0.32–0.81)	0.007	3.157	0.63 (0.20)	0.62 (0.48–0.79)	0.001	3.018	1.046	0.563–1.570

*CI* confidence intervals, *ES* effect size, *RE* relative efficiency

^a^Student’s *t* test compared to the healthy subgroup, where *p* < 0.05 was considered statistically significant

^b^Relative efficiency compared to 15D

^c^2000 bootstrap samples with accelerated bias correction

^d^Includes gambling or other addictions

**Table 6:** Known-groups validity of the EQ-5D-5L (Hungarian value set) and 15D (Norwegian value set)

	n (%)	EQ-5D-5L				15D				RE^b^	95% CI^c^
		Mean (SD)	Median (Q1–Q3)	*p* value^a^	Cohen’s d ES	Mean (SD)	Median (Q1–Q3)	*p* value^a^	Cohen’s d ES		
Healthy	383 (20.3)	0.95 (0.10)	1.0 (0.96–1.00)	–	–	0.90 (0.20)	0.97 (0.89 to 1.00)	–	–	–	–
Physical conditions											
Hypertension	527 (27.9)	0.80 (0.27)	0.89 (0.76–0.97)	< 0.001	1.233	0.75 (0.22)	0.80 (0.63 to 0.93)	< 0.001	1.064	1.031	0.863–1.295
Musculoskeletal diseases	461 (24.4)	0.75 (0.28)	0.84 (0.71–0.92)	< 0.001	1.366	0.71 (0.22)	0.76 (0.59 to 0.89)	< 0.001	1.190	1.054	0.900–1.280
Allergies	318 (16.9)	0.84 (0.22)	0.92 (0.80–1.00)	< 0.001	1.596	0.76 (0.22)	0.82 (0.65 to 0.93)	< 0.001	1.410	1.022	0.818–1.354
Cardiovascular disease	259 (13.7)	0.72 (0.30)	0.83 (0.63–0.92)	< 0.001	2.592	0.65 (0.22)	0.68 (0.48 to 0.84)	< 0.001	2.303	1.002	0.855–1.195
Gastrointestinal or hepatic disease	241 (12.8)	0.77 (0.28)	0.88 (0.71–0.96)	< 0.001	2.685	0.69 (0.22)	0.75 (0.57 to 0.89)	< 0.001	2.386	1.013	0.848–1.252
Hyperlipidaemia	240 (12.7)	0.79 (0.28)	0.88 (0.76–0.96)	< 0.001	1.655	0.71 (0.22)	0.76 (0.58 to 0.89)	< 0.001	1.468	0.977	0.810–1.221
Eye or visual diseases	231 (12.2)	0.76 (0.27)	0.84 (0.70–0.93)	< 0.001	2.739	0.64 (0.22)	0.65 (0.49 to 0.86)	< 0.001	2.434	0.902	0.763–1.083
Diabetes	205 (10.9)	0.77 (0.31)	0.88 (0.71–0.97)	< 0.001	1.890	0.72 (0.22)	0.77 (0.59 to 0.93)	< 0.001	1.684	1.146	0.938–1.483
Gastroesophageal reflux disease	186 (9.9)	0.77 (0.29)	0.88 (0.71–0.96)	< 0.001	1.886	0.71 (0.22)	0.76 (0.57 to 0.90)	< 0.001	1.682	1.128	0.917–1.455
Respiratory diseases	175 (9.3)	0.80 (0.28)	0.89 (0.77–0.96)	< 0.001	3.099	0.70 (0.22)	0.75 (0.57 to 0.89)	< 0.001	2.751	0.933	0.753–1.196
Arrhythmias	172 (9.1)	0.74 (0.26)	0.83 (0.66–0.92)	< 0.001	1.826	0.64 (0.22)	0.67 (0.47 to 0.83)	< 0.001	1.629	1.066	0.880–1.311
Thyroid diseases	171 (9.1)	0.80 (0.26)	0.89 (0.76–0.96)	< 0.001	1.726	0.74 (0.22)	0.79 (0.65 to 0.91)	< 0.001	1.538	1.185	0.954–1.583
Skin diseases	166 (8.8)	0.80 (0.29)	0.92 (0.79–0.96)	< 0.001	3.167	0.72 (0.22)	0.78 (0.59 to 0.92)	< 0.001	2.811	1.007	0.815–1.298
Headache, migraine	139 (7.4)	0.75 (0.31)	0.86 (0.70–0.96)	< 0.001	3.396	0.67 (0.22)	0.74 (0.52 to 0.90)	< 0.001	3.008	1.090	0.890–1.374
Hearing impairment	133 (7.1)	0.75 (0.30)	0.85 (0.65–0.96)	< 0.001	2.332	0.68 (0.22)	0.76 (0.54 to 0.87)	< 0.001	2.086	1.171	0.957–1.487
Benign prostate hyperplasia	88 (4.7)	0.81 (0.24)	0.89 (0.80–0.96)	< 0.001	2.276	0.71 (0.22)	0.76 (0.59 to 0.89)	< 0.001	2.040	1.120	0.832–1.515
Urinary incontinence	71 (3.8)	0.67 (0.34)	0.80 (0.53–0.93)	< 0.001	2.676	0.62 (0.22)	0.68 (0.42 to 0.80)	< 0.001	2.396	1.302	1.049–1.651
Cancer, leukemia, lymphoma	46 (2.4)	0.75 (0.30)	0.88 (0.62–0.96)	< 0.001	2.473	0.66 (0.22)	0.72 (0.48 to 0.86)	< 0.001	2.219	1.300	0.958–1.835
Chronic kidney disease	29 (1.5)	0.73 (0.30)	0.83 (0.58–0.92)	< 0.001	2.690	0.64 (0.22)	0.68 (0.52 to 0.82)	< 0.001	2.412	1.456	1.028–2.074
Epilepsy	17 (0.9)	0.69 (0.35)	0.83 (0.59–0.96)	0.006	3.069	0.58 (0.22)	0.57 (0.38 to 0.85)	0.006	2.745	1.428	0.891–2.240
Liver cirrhosis	14 (0.7)	0.68 (0.38)	0.79 (0.56–0.99)	0.018	2.460	0.56 (0.22)	0.54 (0.36 to 0.89)	0.018	2.210	1.407	0.996–1.903
Other physical health conditions	92 (4.9)	0.76 (0.27)	0.85 (0.65–0.96)	< 0.001	3.029	0.72 (0.22)	0.77 (0.58 to 0.86)	< 0.001	2.701	1.454	1.105–1.962
Mental conditions											
Smoking addiction	381 (20.2)	0.83 (0.25)	0.92 (0.80–0.97)	< 0.001	1.563	0.75 (0.22)	0.82 (0.65 to 0.93)	< 0.001	1.376	1.003	0.824–1.297
Anxiety, phobia, or panic disorder	172 (9.1)	0.71 (0.29)	0.80 (0.61–0.92)	< 0.001	3.121	0.57 (0.22)	0.62 (0.37 to 0.79)	< 0.001	2.771	0.934	0.790–1.128
Sleeping disorders	169 (9.0)	0.70 (0.30)	0.80 (0.62–0.91)	< 0.001	1.809	0.61 (0.22)	0.66 (0.42 to 0.82)	< 0.001	1.613	1.068	0.887–1.302
Other addictions^d^	98 (5.2)	0.79 (0.26)	0.88 (0.77–0.96)	< 0.001	4.360	0.68 (0.22)	0.76 (0.56 to 0.92)	< 0.001	3.816	1.156	0.847–1.584
Depression or dysthymia	79 (4.2)	0.62 (0.32)	0.70 (0.50–0.85)	< 0.001	4.089	0.50 (0.22)	0.55 (0.33 to 0.67)	< 0.001	3.594	1.072	0.861–1.334
Alcohol addiction	73 (3.9)	0.79 (0.26)	0.89 (0.69–0.96)	< 0.001	2.526	0.63 (0.22)	0.72 (0.47 to 0.84)	< 0.001	2.264	0.988	0.723–1.322
Substance addiction	55 (2.9)	0.70 (0.36)	0.88 (0.57–0.96)	0.002	3.027	0.58 (0.22)	0.72 (0.26 to 0.93)	0.002	2.708	1.354	0.833–2.134
Sexual disorder	40 (2.1)	0.74 (0.29)	0.83 (0.65–0.96)	< 0.001	1.649	0.57 (0.22)	0.63 (0.42 to 0.73)	< 0.001	1.475	1.017	0.738–1.330
Bipolar depression	35 (1.9)	0.67 (0.30)	0.76 (0.53–0.87)	< 0.001	1.722	0.53 (0.22)	0.58 (0.32 to 0.73)	< 0.001	1.542	1.253	0.933–1.647
Personality disorder	31 (1.6)	0.61 (0.30)	0.70 (0.45–0.83)	< 0.001	2.096	0.42 (0.22)	0.46 (0.26 to 0.64)	< 0.001	1.883	1.197	0.898–1.596
Learning disability	28 (1.5)	0.76 (0.30)	0.88 (0.64–0.97)	0.002	2.280	0.60 (0.22)	0.73 (0.31 to 0.93)	0.002	2.048	1.145	0.710–1.696
Eating disorder	26 (1.4)	0.69 (0.35)	0.83 (0.59–0.92)	< 0.001	2.288	0.56 (0.22)	0.69 (0.22 to 0.83)	< 0.001	2.055	1.251	0.886–1.702
Obsessive compulsive disorder	21 (1.1)	0.58 (0.35)	0.76 (0.50–0.83)	< 0.001	1.822	0.36 (0.22)	0.44 (0.08 to 0.63)	< 0.001	1.634	1.157	0.736–1.703
Dementia	18 (1.0)	0.47 (0.29)	0.50 (0.25–0.62)	< 0.001	2.229	0.37 (0.22)	0.33 (0.11 to 0.58)	< 0.001	2.004	1.672	1.222–2.373
Psychotic disorders	17 (0.9)	0.66 (0.38)	0.76 (0.57–0.91)	0.005	2.257	0.37 (0.22)	0.33 (0.06 to 0.67)	0.005	2.029	0.972	0.507–1.562
Post-traumatic stress disorder	14 (0.7)	0.55 (0.27)	0.58 (0.51–0.73)	< 0.001	1.725	0.30 (0.22)	0.34 (0.06 to 0.48)	< 0.001	1.546	1.252	0.845–1.709
Impulse-control disorder	14 (0.7)	0.64 (0.36)	0.71 (0.42–0.95)	0.006	2.220	0.42 (0.22)	0.32 (0.25 to 0.66)	0.006	1.995	1.142	0.646–1.765
Autism spectrum disorder	11 (0.6)	0.57 (0.33)	0.70 (0.45–0.82)	0.003	2.536	0.31 (0.22)	0.36 (− 0.02 to 0.60)	0.003	2.278	1.216	0.699–1.888
Attention deficit hyperactivity disorder	10 (0.5)	0.55 (0.35)	0.64 (0.34–0.81)	0.005	2.335	0.29 (0.22)	0.24 (0.01 to 0.57)	0.005	2.099	1.230	0.706–1.816

*CI* confidence interval, *ES* effect size, *RE* relative efficiency

^a^Student’s *t* test compared to the healthy subgroup, where *p* < 0.05 was considered statistically significant

^b^Relative efficiency compared to 15D

^c^2000 bootstrap samples with accelerated bias correction

^d^Includes gambling or other addictions

### Subgroup analysis

The subgroup analysis for the physical and mental health condition subgroups yielded similar results to those of the total sample. Lower ceiling was observed both in the mental (18.7%) and physical health conditions subgroups (25.5%) compared to the total sample (36.0%) for the EQ-5D-5L, while the floor was 0% in both subgroups. Similarly, for the 15D, the ceiling was reduced to a greater extent in the mental health condition subgroup (10.1%) than in the physical health condition subgroup (12.3%) against the total sample (21.0%) (Table 1). In line with previous results, J′ was greater for the EQ-5D-5L than for the 15D in both subgroups (Table 2). The average size of inconsistency was similar for physical and mental health conditions (Additional file 1: Supplementary materials 10–11). The correlation between the Danish EQ-5D-5L and Danish 15D index values was higher in both the physical and mental health condition subgroups (0.736 and 0.702) than in the total sample (0.671). The ICC stood at 0.311 (95% CI 0.285–0.338, *p* < 0.001) for the physical health conditions subgroup, while reached 0.336 (95% CI 0.302–0.371, *p* < 0.001) for the mental health subgroup. As for the corresponding dimensions, correlations between dimensions were, in general, higher in both subgroups than in the total sample (Additional file 1: Supplementary materials 12–13). The sensitivity analyses (Additional file 1: Supplementary materials 6–9, 12–13) with the Hungarian EQ-5D-5L and Norwegian 15D value sets mostly supported these results; however, the agreement was good in both the physical (ICC = 0.653, 95% CI 0.561–0.722, *p* < 0.001) and mental (ICC = 0.632, 95% CI 0.495–0.725, *p* < 0.001) health condition subgroups.

## Discussion

To our knowledge, this is the first study to compare the measurement properties of the EQ-5D-5L and 15D instruments in a general population sample. The sample showed good representativeness across demographic characteristics and allowed conducting subgroup analyses for physical and mental health conditions. EQ-5D-5L dimensions showed a substantially lower ceiling than those of the 15D in all but one corresponding dimension pairs. We identified a considerably larger ceiling in the EQ-5D-5L index value than the 15D index value, which corroborates earlier findings in various patient populations [26, 28, 29, 31, 32]. The ceiling decreased notably in both the physical and mental conditions subgroups compared to the total sample concerning both indices. The EQ-5D-5L demonstrated better overall relative informativity. Strong correlations were seen between the index values, which can be confirmed by previous research [30, 31]. Differently from our expectations [56], the anxiety/depression composite dimension correlated stronger with 15D depression than with 15D distress. Both the EQ-5D-5L and 15D were able to discriminate effectively between the healthy and non-healthy respondents with moderate or large effect sizes; however, EQ-5D-5L produced larger effect sizes in most groups regardless of the value set used.

Both instruments were able to effectively discriminate between the healthy and non-healthy groups of respondents. However, it is worth mentioning that although the index values in the healthy subgroup were reasonably similar for both the EQ-5D-5L and 15D using the Danish value sets, the mean index values of the EQ-5D-5L were substantially lower than those for the 15D in respondents with health conditions. On the contrary, the sensitivity analysis suggests that the Norwegian 15D index values were sizeably lower in 15/41 health conditions than the Hungarian EQ-5D-5L index values, while the difference was negligible in the rest. This is mainly attributable to the different value sets of the 15D. The range of the Danish value set is considerably narrower than that of the Norwegian, which has a utility of − 0.516 for the worst possible state that is more comparable to either EQ-5D-5L value sets used in this study. Therefore, there is less space for potential improvement using the Danish 15D value set and for this reason, the index values of more severe health states are already relatively high. The difference between the value sets is also well indicated by the fact that the ICC is poor between the Danish EQ-5D-5L and 15D index values, but good between the Hungarian EQ-5D-5L and Norwegian 15D.

A few 15D dimensions demonstrated (very) weak correlations with all EQ-5D-5L dimensions, such as vision, hearing, eating, speech, excretion, and mental function, which may indicate potential room for EQ-5D-5L bolt-ons. This is in line with earlier research that acknowledged these health areas as potentially not captured by the EQ-5D and proposed bolt-ons for these, including vision, hearing, speech, and cognition [34, 37, 57–59]. As bolt-on identification, development and testing are recommended to be based on mixed-methods evidence from multiple investigations and populations [36], our results support these efforts by informing future EQ-5D bolt-on development studies.

The following limitations should be considered. Firstly, due to the cross-sectional design of our study, we could not test the responsiveness or the test–retest reliability of the instruments. Secondly, according to census data, 48.0% of the Hungarian general population reported having chronic illness [38], whereas in our sample this proportion reached 71.6%. This difference is likely due to the fact that our questionnaire was rather detailed regarding questions about different health conditions and considered addictions (e.g. smoking) as well. Thirdly, clinical data including information on disease severity were not available from our survey, which would have allowed a more comprehensive known-groups validity testing. Finally, we have to acknowledge some linguistic specificities of our findings. For instance, in English, the mobility dimensions of both instruments use the phrase ‘walking’, while the Hungarian version of the 15D uses a different translation with a meaning of ‘moving about’ (‘közlekedés’) that could be responsible for the relatively high proportion of inconsistent response pairs between these two dimensions (7.9%).

## Conclusions

In conclusion, our findings may contribute to the discussion of which generic PAM to use in decision-making and provide useful and broad information for health economic evaluations. Despite having 10 fewer dimensions, the EQ-5D-5L performed better than the 15D in many aspects. However, certain 15D dimensions (e.g. vision, hearing, mental function) showed a relatively weak relationship with the dimensions of EQ-5D-5L, which signals room for potential EQ-5D-5L bolt-on dimensions. Future research is recommended to assess the added value of such bolt-on dimensions and compare their measurement properties to other PAMs that include these health areas among their dimensions (e.g. 15D, AQoL). Additionally, longitudinal studies are needed to test the responsiveness of these instruments in relevant patient populations.

## Supplementary Information

**Additional file 1.** Supplementary materials.

## Data Availability

All data of this study are available from the corresponding author upon reasonable request.

## Abbreviations

AQoL: Assessment of Quality of Life
CI: Confidence interval
cTTO: Composite time trade-off
DCE: Discrete choice experiment
DSM-5: Fifth edition of the Diagnostic and Statistical Manual of Mental Disorders
EHIS: European Health Interview Survey
ES: Effect size
H′: Shannon’s index
HUI: Health Utilities Index
ICC: Intraclass correlation coefficient
J′: Shannon’s Evenness index
PAM: Preference-accompanied measure
QALY: Quality Adjusted Life Years
RE: Relative efficiency
SD: Standard deviation
SF-6D: Short-Form 6-Dimension
VAS: Visual analogue scale

## References

[1] Finch AP, Brazier JE, Mukuria C (2018). What is the evidence for the performance of generic preference-based measures? A systematic overview of reviews. Eur J Health Econ.

[2] Kennedy-Martin M, Slaap B, Herdman M, van Reenen M, Kennedy-Martin T, Greiner W (2020). Which multi-attribute utility instruments are recommended for use in cost-utility analysis? A review of national health technology assessment (HTA) guidelines. Eur J Health Econ.

[3] Rencz F, Gulácsi L, Drummond M, Golicki D, Prevolnik Rupel V, Simon J (2016). EQ-5D in central and eastern Europe: 2000–2015. Qual Life Res.

[4] Richardson J, Iezzi A, Khan MA (2015). Why do multi-attribute utility instruments produce different utilities: the relative importance of the descriptive systems, scale and 'micro-utility' effects. Qual Life Res.

[5] The EuroQol Group (1990). EuroQol—a new facility for the measurement of health-related quality of life. Health Policy.

[6] Herdman M, Gudex C, Lloyd A, Janssen M, Kind P, Parkin D (2011). Development and preliminary testing of the new five-level version of EQ-5D (EQ-5D-5L). Qual Life Res.

[7] Feng YS, Kohlmann T, Janssen MF, Buchholz I (2021). Psychometric properties of the EQ-5D-5L: a systematic review of the literature. Qual Life Res.

[8] Longworth L, Singh J, Brazier J (2014). An evaluation of the performance of EQ-5D: a review of reviews of psychometric properties. Value Health.

[9] Sintonen H (2001). The 15D instrument of health-related quality of life: properties and applications. Ann Med.

[10] Sintonen H. 15D instrument. http://www.15d-instrument.net/15d/. Accessed 24 May 2022.

[11] Sintonen H. The 15D-measure of health-related quality of life. II. Feasibility, reliability and validity of its valuation system. National Centre for Health Program Evaluation, Working Paper 42, Melbourne. 1995.

[12] Wittrup-Jensen KU, Pedersen KM (2008). Modelling Danish weights for the 15D quality of life questionnaire by applying multi-attribute utility theory (MAUT).

[13] Michel YA, Augestad LA, Rand K (2018). Comparing 15D valuation studies in Norway and Finland-challenges when combining information from several valuation tasks. Value Health.

[14] Michel YA, Augestad LA, Barra M, Rand K (2019). A Norwegian 15D value algorithm: proposing a new procedure to estimate 15D value algorithms. Qual Life Res.

[15] Norwegian Medicines Agency. Guidelines for the submission of documentation for single technology assessment (STA) of pharmaceuticals. https://legemiddelverket.no/Documents/English/Public%20funding%20and%20pricing/Documentation%20for%20STA/Guidelines%20151018.pdf. Published 2018. Accessed 6 Dec 2022.

[16] Ministerio de Salud de Chile. Guía Metodológica para la Evaluación Económica de Intervenciones en Salud en Chile [Methodological Guide for the Economic Evaluation of Health Interventions in Chile]. https://www.orasconhu.org/case/sites/default/files/files/EE_FINAL_web.pdf. Published 2013. Accessed 6 Dec 2022.

[17] Rissanen P, Aro S, Sintonen H, Asikainen K, Slätis P, Paavolainen P (1997). Costs and cost-effectiveness in hip and knee replacements. A prospective study. Int J Technol Assess Health Care.

[18] Linna M, Taimela E, Apajasalo M, Marttila RJ (2002). Probabilistic sensitivity analysis for evaluating cost-utility of entacapone for Parkinson's disease. Expert Rev Pharmacoecon Outcomes Res.

[19] Räsänen P, Krootila K, Sintonen H, Leivo T, Koivisto AM, Ryynänen OP (2006). Cost-utility of routine cataract surgery. Health Qual Life Outcomes.

[20] Kantola T, Mäklin S, Koivusalo AM, Räsänen P, Rissanen A, Roine R (2010). Cost-utility of molecular adsorbent recirculating system treatment in acute liver failure. World J Gastroenterol.

[21] Åberg F, Mäklin S, Räsänen P, Roine RP, Sintonen H, Koivusalo AM (2011). Cost of a quality-adjusted life year in liver transplantation: the influence of the indication and the model for end-stage liver disease score. Liver Transpl.

[22] Pohjolainen V, Räsänen P, Roine RP, Sintonen H, Koponen S, Karlsson H (2017). Cost-effectiveness of anorexia nervosa in QALYs. Nord J Psychiatry.

[23] Gulbrandsen N, Wisløff F, Nord E, Lenhoff S, Hjorth M, Westin J (2001). Cost-utility analysis of high-dose melphalan with autologous blood stem cell support vs. melphalan plus prednisone in patients younger than 60 years with multiple myeloma. Eur J Haematol..

[24] Ross S, Robert M, Lier D, Eliasziw M, Jacobs P (2011). Surgical management of stress urinary incontinence in women: safety, effectiveness and cost-utility of trans-obturator tape (TOT) versus tension-free vaginal tape (TVT) five years after a randomized surgical trial. BMC Womens Health.

[25] Bastani P, Kiadaliri AA (2012). Cost-utility analysis of adjuvant therapies for breast cancer in Iran. Int J Technol Assess Health Care.

[26] Stavem K, Bjørnaes H, Lossius MI (2001). Properties of the 15D and EQ-5D utility measures in a community sample of people with epilepsy. Epilepsy Res.

[27] Stavem K, Frøland SS, Hellum KB (2005). Comparison of preference-based utilities of the 15D, EQ-5D and SF-6D in patients with HIV/AIDS. Qual Life Res.

[28] Lunde L (2013). Can EQ-5D and 15D be used interchangeably in economic evaluations? Assessing quality of life in post-stroke patients. Eur J Health Econ.

[29] Saarni SI, Härkänen T, Sintonen H, Suvisaari J, Koskinen S, Aromaa A (2006). The impact of 29 chronic conditions on health-related quality of life: a general population survey in Finland using 15D and EQ-5D. Qual Life Res.

[30] Vartiainen P, Mäntyselkä P, Heiskanen T, Hagelberg N, Mustola S, Forssell H (2017). Validation of EQ-5D and 15D in the assessment of health-related quality of life in chronic pain. Pain.

[31] Vainiola T, Pettilä V, Roine RP, Räsänen P, Rissanen AM, Sintonen H (2010). Comparison of two utility instruments, the EQ-5D and the 15D, in the critical care setting. Intensive Care Med.

[32] Heiskanen J, Tolppanen AM, Roine RP, Hartikainen J, Hippeläinen M, Miettinen H (2016). Comparison of EQ-5D and 15D instruments for assessing the health-related quality of life in cardiac surgery patients. Eur Heart J Qual Care Clin Outcomes.

[33] García-Gordillo M, del Pozo-Cruz B, Adsuar JC, Sánchez-Martínez FI, Abellán-Perpiñán JM (2014). Validation and comparison of 15-D and EQ-5D-5L instruments in a Spanish Parkinson's disease population sample. Qual Life Res.

[34] Longworth L, Yang Y, Young T, Mulhern B, Hernández Alava M, Mukuria C (2014). Use of generic and condition-specific measures of health-related quality of life in NICE decision-making: a systematic review, statistical modelling and survey. Health Technol Assess.

[35] Brazier J (2010). Is the EQ-5D fit for purpose in mental health?. Br J Psychiatry.

[36] Mulhern BJ, Sampson C, Haywood P, Addo R, Page K, Mott D (2022). Criteria for developing, assessing and selecting candidate EQ-5D bolt-ons. Qual Life Res..

[37] Geraerds A, Bonsel GJ, Janssen MF, Finch AP, Polinder S, Haagsma JA (2021). Methods used to identify, test, and assess impact on preferences of bolt-ons: a systematic review. Value Health.

[38] Hungarian Central Statistical Office. Tehetünk az egészségünkért – ELEF2019 gyorsjelentés. https://www.ksh.hu/docs/hun/xftp/idoszaki/elef/te_2019/index.html. Accessed 11 March 2022.

[39] American Psychiatric Association. Diagnostic and statistical manual of mental disorders. 5th ed. Washington, DC; 2013.

[40] Jensen CE, Sørensen SS, Gudex C, Jensen MB, Pedersen KM, Ehlers LH (2021). The Danish EQ-5D-5L value set: a hybrid model using cTTO and DCE data. Appl Health Econ Health Policy.

[41] Roudijk B, Donders ART, Stalmeier PFM (2019). Cultural values: can they explain differences in health utilities between countries?. Med Decis Making.

[42] Rencz F, Brodszky V, Gulácsi L, Golicki D, Ruzsa G, Pickard AS (2020). Parallel valuation of the EQ-5D-3L and EQ-5D-5L by time trade-off in Hungary. Value Health.

[43] Rencz F, Brodszky V, Janssen MF. A direct comparison of the measurement properties of EQ-5D-5L, PROMIS-29+2 and PROMIS Global Health instruments and EQ-5D-5L and PROPr utilities in a general population sample. Value Health. 2023 [In press] 10.1016/j.jval.2023.02.002 36804583

[44] Brazier J, Roberts J, Tsuchiya A, Busschbach J (2004). A comparison of the EQ-5D and SF-6D across seven patient groups. Health Econ.

[45] Janssen MF, Bonsel GJ, Luo N (2018). Is EQ-5D-5L better than EQ-5D-3L? A head-to-head comparison of descriptive systems and value sets from seven countries. Pharmacoeconomics.

[46] Janssen MFB, Birnie E, Bonsel GJ (2007). Evaluating the discriminatory power of EQ-5D, HUI2 and HUI3 in a US general population survey using Shannon's indices. Qual Life Res.

[47] Shannon CE (1948). A mathematical theory of communication. Bell Syst Tech J.

[48] Shannon C, Weaver W (1949). The mathematical theory of communication.

[49] Janssen MF, Birnie E, Haagsma JA, Bonsel GJ (2008). Comparing the standard EQ-5D three-level system with a five-level version. Value Health.

[50] Koo TK, Li MY (2016). A Guideline of selecting and reporting intraclass correlation coefficients for reliability research. J Chiropr Med.

[51] Bland JM, Altman DG (1986). Statistical methods for assessing agreement between two methods of clinical measurement. Lancet.

[52] Shrout PE, Fleiss JL (1979). Intraclass correlations: uses in assessing rater reliability. Psychol Bull.

[53] Cicchetti DV (1994). Guidelines, criteria, and rules of thumb for evaluating normed and standardized assessment instruments in psychology. Psychol Assess.

[54] Swinscow TDV, Campbell MJ (2002). Statistics at square one.

[55] Cohen J (1992). A power primer. Psychol Bull.

[56] Rencz F, Janssen MF (2022). Analyzing the pain/discomfort and anxiety/depression composite domains and the meaning of discomfort in the EQ-5D: a mixed-methods study. Value Health.

[57] Krabbe PF, Stouthard ME, Essink-Bot ML, Bonsel GJ (1999). The effect of adding a cognitive dimension to the EuroQol multiattribute health-status classification system. J Clin Epidemiol.

[58] Finch AP, Brazier JE, Mukuria C, Bjorner JB (2017). An Exploratory study on using principal-component analysis and confirmatory factor analysis to identify bolt-on dimensions: the EQ-5D case study. Value Health.

[59] Finch AP, Brazier JE, Mukuria C (2019). Selecting bolt-on dimensions for the EQ-5D: examining their contribution to health-related quality of life. Value Health.

