# Supplementary material for: A head-to-head comparison of the EQ-5D-5L and 15D descriptive systems and index values in a general population sample
Source: Health Qual Life Outcomes. 2023 Feb 19;21:17. doi: 10.1186/s12955-023-02096-z (PMC9940337; doi:10.1186/s12955-023-02096-z)
Supplement: Supplementary file 1 — Additional file 1. Supplementary materials. [file 12955_2023_2096_MOESM1_ESM.docx]

**Supplementary Materials**

**Title:** A head-to-head comparison of the EQ-5D-5L and 15D descriptive systems and index values in a general population sample

**Authors:** Anna Nikl, Mathieu F. Janssen, Valentin Brodszky and Fanni Rencz

**Journal:** Health and Quality of Life Outcomes

**Correspondence:**

Fanni Rencz

Department of Health Policy

Corvinus University of Budapest

8 Fővám tér, H-1093 Budapest, Hungary

fanni.rencz@uni-corvinus.hu

**Supplementary material 1 Characteristics of the study population**

| Variables | Total sample (N=1887) | | Physical conditions (N=1195) ^a^ | | Mental conditions (N=664) ^a^ | | Hungarian general population (%) (1, 2) | Proportional difference to the total sample (pp) |
| --- | --- | --- | --- | --- | --- | --- | --- | --- |
|  | n | % | n | % | n | % |  |  |
| Gender | | | | | | | | |
| Male | 800 | 42.4 | 486 | 40.7 | 271 | 40.8 | 46.9 | -4.5 |
| Female | 1087 | 57.6 | 709 | 59.3 | 393 | 59.2 | 53.1 | 4.5 |
| Age groups, years | | | | | | | | |
| 18-24 | 191 | 10.1 | 67 | 5.6 | 59 | 8.9 | 10.0 | 0.1 |
| 25-34 | 413 | 21.9 | 196 | 16.4 | 140 | 21.1 | 15.2 | 6.7 |
| 35-44 | 309 | 16.4 | 163 | 13.6 | 106 | 16.0 | 19.5 | -3.1 |
| 45-54 | 266 | 14.1 | 184 | 15.4 | 97 | 14.6 | 16.0 | -1.9 |
| 55-64 | 318 | 16.9 | 249 | 20.8 | 123 | 18.5 | 16.8 | 0.1 |
| 65 and above | 390 | 20.7 | 336 | 28.1 | 139 | 20.9 | 22.5 | -1.8 |
| Settlement type | | | | | | | | |
| Capital | 367 | 19.4 | 223 | 18.7 | 110 | 16.6 | 17.9 | 1.5 |
| City | 923 | 48.9 | 557 | 48.3 | 313 | 47.1 | 52.6 | -3.7 |
| Village | 597 | 31.6 | 395 | 33.1 | 241 | 36.3 | 29.5 | 2.1 |
| Geographical region ^b^ | | | | | | | | |
| Central Hungary | 582 | 30.8 | 366 | 30.6 | 202 | 30.4 | 30.7 | 0.1 |
| Transdanubia | 559 | 29.6 | 364 | 30.5 | 201 | 30.3 | 30.1 | -0.5 |
| Great Plain and North | 746 | 39.5 | 465 | 38.9 | 261 | 39.3 | 39.2 | 0.3 |
| Highest level of education | | | | | | | | |
| Primary | 508 | 26.9 | 323 | 27.0 | 218 | 32.8 | 23.8 | 3.1 |
| Secondary | 846 | 44.8 | 518 | 43.3 | 305 | 45.9 | 55.0 | -10.2 |
| Tertiary | 533 | 28.2 | 354 | 29.6 | 141 | 21.2 | 21.2 | 7 |
| Marital status | | | | | | | | |
| Single | 444 | 23.5 | 228 | 19.1 | 150 | 22.6 | 18.5 | 5 |
| Married | 789 | 41.8 | 541 | 45.3 | 247 | 37.2 | 45.6 | -3.8 |
| Divorced | 142 | 7.5 | 114 | 9.5 | 69 | 10.4 | 11.1 | -3.6 |
| Widowed | 122 | 6.5 | 98 | 8.2 | 46 | 6.9 | 11.4 | -4.9 |
| Domestic partnership | 390 | 20.7 | 214 | 17.9 | 152 | 22.9 | 13.4 | 7.3 |
| Occupational status | | | | | | | | |
| Employed | 1000 | 53.0 | 565 | 47.3 | 340 | 51.2 | 53.1 | -0.1 |
| Unemployed | 89 | 4.7 | 45 | 3.8 | 38 | 5.7 | 3.1 | 1.6 |
| Retired | 489 | 25.9 | 405 | 33.9 | 171 | 25.8 | 26.1 | -0.2 |
| Disability pensioner | 52 | 2.8 | 48 | 4.0 | 28 | 4.2 | 3.1 | -0.3 |
| Student | 64 | 3.4 | 22 | 1.8 | 12 | 1.8 | 4.7 | -1.3 |
| Stay-at-home husband/wife | 48 | 2.5 | 30 | 2.5 | 29 | 4.4 | 1.0 | 1.5 |
| Other | 145 | 7.7 | 80 | 6.7 | 46 | 6.9 | 8.9 | -1.2 |
| Diagnosed chronic disease | | | | | | | | |
| Mental | 155 | 8.2 | - | - | 155 | 23.3 | N/A | - |
| Physical | 686 | 36.4 | 686 | 57.4 | - | - | 48.0 ^c^ | -15.4 |
| Both | 509 | 27.0 | 509 | 42.6 | 509 | 76.7 |  |  |
| None | 383 | 20.3 | - | - | - | - | 52.0 ^c^ | 31.7 |
| Does not know/answer | 154 | 8.2 | - | - | - | - | N/A | - |

N/A indicates data not available. pp: percentage points.

Percentages may not total 100 by groups due to rounding.

^a^ There are overlaps between the physical and mental conditions subgroups as n=509 respondents reported to have both physical and mental conditions.

^b^ Figure represents the population aged 15 or over for the general population

^c^ General population sample aged 15 or over, 2019 European Health Interview Survey in Hungary (2)

Supplementary material 2 Cross-tabulation of EQ-5D-5L and 15D responses between the corresponding dimensions

| EQ-5D-5L | 15D | | | | | Inconsistent response pairs, n (%) | Average size of inconsistencies |
| --- | --- | --- | --- | --- | --- | --- | --- |
| Dimensions | Level 1 | Level 2 | Level 3 | Level 4 | Level 5 |  |  |
| Mobility, n (%) | | | | | | |  |
| Level 1 | 1163 (93.3) | 49 (3.9) | 27 (2.2) | 1 (0.1) | 6 (0.5) | 149 (7.90) | 1.20 |
| Level 2 | 223 (64.1) | 108 (31.0) | 14 (4.0) | 1 (0.3) | 2 (0.6) |  |  |
| Level 3 | 71 (35.9) | 98 (49.5) | 20 (10.1) | 6 (3.0) | 3 (1.5) |  |  |
| Level 4 | 15 (17.0) | 22 (25.0) | 48 (54.5) | 3 (3.4) | 0 (0.0) |  |  |
| Level 5 | 0 (0.0) | 0 (0.0) | 1 (14.3) | 3 (42.9) | 3 (42.9) |  |  |
| Usual activities, n (%) | | | | | | |  |
| Level 1 | 1290 (92.6) | 73 (5.2) | 23 (1.7) | 5 (0.4) | 2 (0.1) | 88 (4.66) | 1.24 |
| Level 2 | 136 (45.2) | 138 (45.8) | 23 (7.6) | 4 (1.3) | 0 (0.0) |  |  |
| Level 3 | 29 (22.1) | 63 (48.1) | 29 (22.1) | 9 (6.9) | 1 (0.8) |  |  |
| Level 4 | 12 (20.7) | 12 (20.7) | 16 (27.6) | 15 (25.9) | 3 (5.2) |  |  |
| Level 5 | 0 (0.0) | 0 (0.0) | 0 (0.0) | 2 (50.0) | 2 (50.0) |  |  |
| Pain/discomfort (EQ-5D-5L) *and* Discomfort and symptoms (15D), n (%) | | | | | | |  |
| Level 1 | 884 (92.2) | 59 (6.2) | 9 (0.9) | 5 (0.5) | 2 (0.2) | 122 (6.47) | 1.16 |
| Level 2 | 322 (54.7) | 231 (39.2) | 32 (5.4) | 2 (0.3) | 2 (0.3) |  |  |
| Level 3 | 72 (27.2) | 112 (42.3) | 67 (25.3) | 12 (4.5) | 2 (0.8) |  |  |
| Level 4 | 9 (13.8) | 18 (27.7) | 18 (27.7) | 18 (27.7) | 2 (3.1) |  |  |
| Level 5 | 0 (0.0) | 0 (0.0) | 1 (11.1) | 7 (77.8) | 1 (11.1) |  |  |
| Anxiety/depression (EQ-5D-5L) *and* Depression (15D), n (%) | | | | | | |  |
| Level 1 | 1063 (92.7) | 59 (5.1) | 17 (1.5) | 4 (0.3) | 4 (0.3) | 87 (4.61) | 1.24 |
| Level 2 | 196 (43.6) | 221 (49.1) | 27 (6.0) | 5 (1.1) | 1 (0.2) |  |  |
| Level 3 | 30 (14.5) | 82 (39.6) | 76 (36.7) | 15 (7.2) | 4 (1.9) |  |  |
| Level 4 | 5 (8.3) | 10 (16.7) | 23 (38.3) | 18 (30.0) | 4 (6.7) |  |  |
| Level 5 | 1 (4.3) | 1 (4.3) | 5 (21.7) | 8 (34.8) | 8 (34.8) |  |  |
| Anxiety/depression (EQ-5D-5L) *and* Distress (15D), n (%) | | | | | | |  |
| Level 1 | 911 (79.4) | 201 (17.5) | 24 (2.1) | 9 (0.8) | 2 (0.2) | 102 (5.41) | 1.24 |
| Level 2 | 110 (24.4) | 273 (60.7) | 51 (11.3) | 13 (2.9) | 3 (0.7) |  |  |
| Level 3 | 26 (12.6) | 71 (34.3) | 69 (33.3) | 35 (16.9) | 6 (2.9) |  |  |
| Level 4 | 7 (11.7) | 9 (15.0) | 19 (31.7) | 14 (23.3) | 11 (18.3) |  |  |
| Level 5 | 0 (0.0) | 1 (4.3) | 2 (8.7) | 9 (39.1) | 11 (47.8) |  |  |

Percentages may not total 100 by rows due to rounding.

Supplementary material 3 Distribution of EQ-5D-5L and 15D results within each domain (N=1887)

| EQ-5D-5L | | | | | | 15D | | | | | |
| --- | --- | --- | --- | --- | --- | --- | --- | --- | --- | --- | --- |
| Dimensions | Levels | | | | | Dimensions | Levels | | | | |
|  | 1 | 2 | 3 | 4 | 5 |  | 1 | 2 | 3 | 4 | 5 |
| Mobility (walking) | 1246 (66.0) | 348 (18.4) | 198 (10.5) | 88 (4.7) | 7 (0.4) | Mobility (walking, moving about) | 1472 (78.0) | 277 (14.7) | 110 (5.8) | 14 (0.7) | 14 (0.7) |
| Self-care (washing or dressing) | 1654 (87.7) | 127 (6.7) | 68 (3.6) | 29 (1.5) | 9 (0.5) | - | - | - | - | - | - |
| Usual activities (e.g. work, study, housework, family or leisure activities) | 1393 (73.8) | 301 (16.0) | 131 (6.9) | 58 (3.1) | 4 (0.2) | Usual activities (e.g. employment, studying, housework, free-time activities) | 1467 (77.7) | 286 (15.2) | 91 (4.8) | 35 (1.9) | 8 (0.4) |
| Pain/discomfort | 959 (50.8) | 589 (31.2) | 265 (14.0) | 65 (3.4) | 9 (0.5) | Discomfort and symptoms (e.g. pain, ache, nausea, itching etc.) | 1287 (68.2) | 420 (22.3) | 127 (6.7) | 44 (2.3) | 9 (0.5) |
| Anxiety/depression | 1147 (60.8) | 450 (23.8) | 207 (11.0) | 60 (3.2) | 23 (1.2) | Depression (sad, melancholic or depressed) | 1295 (68.6) | 373 (19.8) | 148 (7.8) | 50 (2.6) | 21 (1.1) |
|  |  |  |  |  |  | Distress (anxious, stressed or nervous) | 1054 (55.9) | 555 (29.4) | 165 (8.7) | 80 (4.2) | 33 (1.7) |
| - | - | - | - | - | - | Vision (seeing and reading with or without glasses) | 1360 (72.1) | 355 (18.8) | 125 (6.6) | 30 (1.6) | 17 (0.9) |
|  |  |  |  |  |  | Hearing (with or without a hearing aid) | 1581 (83.8) | 200 (10.6) | 83 (4.4) | 17 (0.9) | 6 (0.3) |
|  |  |  |  |  |  | Breathing (breathing difficulties, shortness of breath) | 1342 (71.1) | 400 (21.2) | 95 (5.0) | 29 (1.5) | 21 (1.1) |
|  |  |  |  |  |  | Sleeping | 921 (48.8) | 644 (34.1) | 246 (13.0) | 62 (3.3) | 14 (0.7) |
|  |  |  |  |  |  | Eating | 1781 (94.4) | 54 (2.9) | 37 (2.0) | 12 (0.6) | 3 (0.2) |
|  |  |  |  |  |  | Speech | 1701 (90.1) | 124 (6.6) | 44 (2.3) | 13 (0.7) | 5 (0.3) |
|  |  |  |  |  |  | Excretion (bladder and bowel) | 1399 (74.1) | 382 (20.2) | 75 (4.0) | 17 (0.9) | 14 (0.7) |
|  |  |  |  |  |  | Mental function (thinking clearly and logically, memory) | 1596 (84.6) | 218 (11.6) | 48 (2.5) | 18 (1.0) | 7 (0.4) |
|  |  |  |  |  |  | Vitality (e.g., healthy and energetic, weary, tired or feeble, exhausted) | 950 (50.3) | 624 (33.1) | 203 (10.8) | 90 (4.8) | 20 (1.1) |
|  |  |  |  |  |  | Sexual activities | 1313 (69.6) | 300 (15.9) | 155 (8.2) | 46 (2.4) | 73 (3.9) |

Percentages may not total 100 by rows due to rounding.

Supplementary material 4 Distribution of EQ-5D-5L and 15D results within each domain among respondents with physical conditions (N=1195)

| EQ-5D-5L | | | | | | 15D | | | | | |
| --- | --- | --- | --- | --- | --- | --- | --- | --- | --- | --- | --- |
| Dimensions | Levels | | | | | Dimensions | Levels | | | | |
|  | 1 | 2 | 3 | 4 | 5 |  | 1 | 2 | 3 | 4 | 5 |
| Mobility (walking) | 670 (56.1) | 279 (23.2) | 163 (13.6) | 78 (6.5) | 5 (0.4) | Mobility (walking, moving about) | 877 (73.4) | 224 (18.7) | 80 (6.7) | 8 (0.7) | 6 (0.5) |
| Self-care (washing or dressing) | 1027 (85.9) | 91 (7.6) | 47 (3.9) | 22 (1.8) | 8 (0.7) | - | - | - | - | - | - |
| Usual activities (e.g. work, study, housework, family or leisure activities) | 798 (66.8) | 234 (19.6) | 112 (9.4) | 49 (4.1) | 2 (0.2) | Usual activities (e.g. employment, studying, housework, free-time activities) | 857 (71.7) | 248 (20.8) | 61 (5.1) | 28 (2.3) | 1 (0.1) |
| Pain/discomfort | 474 (39.7) | 436 (36.5) | 223 (18.7) | 55 (4.6) | 7 (0.6) | Discomfort and symptoms (e.g. pain, ache, nausea, itching etc.) | 719 (60.2) | 344 (28.8) | 93 (7.8) | 37 (3.1) | 2 (0.2) |
| Anxiety/depression | 675 (56.5) | 309 (25.9) | 149 (12.5) | 46 (3.8) | 16 (1.3) | Depression (sad, melancholic or depressed) | 777 (65.0) | 273 (22.8) | 99 (8.3) | 34 (2.8) | 12 (1.0) |
|  |  |  |  |  |  | Distress (anxious, stressed or nervous) | 607 (50.8) | 397 (33.2) | 114 (9.5) | 58 (4.9) | 19 (1.6) |
| - | - | - | - | - | - | Vision (seeing and reading with or without glasses) | 812 (67.9) | 266 (22.3) | 89 (7.4) | 23 (1.9) | 5 (0.4) |
|  |  |  |  |  |  | Hearing (with or without a hearing aid) | 966 (80.8) | 170 (14.2) | 45 (3.8) | 12 (1.0) | 2 (0.2) |
|  |  |  |  |  |  | Breathing (breathing difficulties, shortness of breath) | 765 (60.4) | 330 (27.6) | 55 (4.6) | 28 (2.3) | 17 (1.4) |
|  |  |  |  |  |  | Sleeping | 491 (41.1) | 462 (38.7) | 178 (14.9) | 55 (4.6) | 9 (0.8) |
|  |  |  |  |  |  | Eating | 1150 (96.2) | 31 (2.6) | 11 (0.9) | 3 (0.3) | 0 (0.0) |
|  |  |  |  |  |  | Speech | 1084 (90.7) | 86 (7.2) | 15 (1.3) | 8 (0.7) | 2 (0.2) |
|  |  |  |  |  |  | Excretion (bladder and bowel) | 814 (68.1) | 314 (26.3) | 51 (4.3) | 9 (0.8) | 7 (0.6) |
|  |  |  |  |  |  | Mental function (thinking clearly and logically, memory) | 989 (82.8) | 172 (14.4) | 27 (2.3) | 6 (0.5) | 1 (0.1) |
|  |  |  |  |  |  | Vitality (e.g., healthy and energetic, weary, tired or feeble, exhausted) | 502 (42.0) | 458 (38.3) | 162 (13.6) | 63 (5.3) | 10 (0.8) |
|  |  |  |  |  |  | Sexual activities | 735 (61.5) | 240 (20.1) | 120 (10.0) | 34 (2.8) | 66 (5.5) |

Percentages may not total 100 by rows due to rounding.

Supplementary material 5 Distribution of EQ-5D-5L and 15D results within each domain among respondents with mental conditions (N=664)

| EQ-5D-5L | | | | | | 15D | | | | | |
| --- | --- | --- | --- | --- | --- | --- | --- | --- | --- | --- | --- |
| Dimensions | Levels | | | | | Dimensions | Levels | | | | |
|  | 1 | 2 | 3 | 4 | 5 |  | 1 | 2 | 3 | 4 | 5 |
| Mobility (walking) | 359 (54.1) | 152 (22.9) | 107 (16.1) | 43 (6.5) | 3 (0.5) | Mobility (walking, moving about) | 467 (70.3) | 137 (20.6) | 51 (7.7) | 7 (1.1) | 2 (0.3) |
| Self-care (washing or dressing) | 538 (81.0) | 72 (10.8) | 34 (5.1) | 16 (2.4) | 4 (0.6) | - | - | - | - | - | - |
| Usual activities (e.g. work, study, housework, family or leisure activities) | 415 (62.5) | 142 (21.4) | 72 (10.8) | 33 (5.0) | 2 (0.3) | Usual activities (e.g. employment, studying, housework, free-time activities) | 436 (65.7) | 148 (22.3) | 52 (7.8) | 27 (4.1) | 1 (0.2) |
| Pain/discomfort | 226 (34.0) | 242 (36.4) | 145 (21.8) | 43 (6.5) | 8 (1.2) | Discomfort and symptoms (e.g. pain, ache, nausea, itching etc.) | 355 (53.5) | 201 (30.3) | 70 (10.5) | 34 (5.1) | 4 (0.6) |
| Anxiety/depression | 272 (41.0) | 205 (30.9) | 134 (20.2) | 37 (5.6) | 16 (2.4) | Depression (sad, melancholic or depressed) | 343 (51.7) | 191 (28.8) | 85 (12.8) | 35 (5.3) | 10 (1.5) |
|  |  |  |  |  |  | Distress (anxious, stressed or nervous) | 262 (39.5) | 229 (34.5) | 101 (15.2) | 54 (8.1) | 18 (2.7) |
| - | - | - | - | - | - | Vision (seeing and reading with or without glasses) | 408 (61.4) | 169 (25.5) | 63 (9.5) | 18 (2.7) | 6 (0.9) |
|  |  |  |  |  |  | Hearing (with or without a hearing aid) | 512 (77.1) | 100 (15.1) | 41 (6.2) | 10 (1.5) | 1 (0.2) |
|  |  |  |  |  |  | Breathing (breathing difficulties, shortness of breath) | 379 (57.1) | 201 (30.3) | 50 (7.5) | 21 (3.2) | 13 (2.0) |
|  |  |  |  |  |  | Sleeping | 225 (33.9) | 234 (35.2) | 142 (21.4) | 54 (8.1) | 9 (1.4) |
|  |  |  |  |  |  | Eating | 608 (91.6) | 30 (4.5) | 19 (2.9) | 7 (1.1) | 0 (0.0) |
|  |  |  |  |  |  | Speech | 564 (84.9) | 69 (10.4) | 19 (2.9) | 10 (1.5) | 2 (0.3) |
|  |  |  |  |  |  | Excretion (bladder and bowel) | 427 (64.3) | 170 (25.6) | 50 (7.5) | 11 (1.7) | 6 (0.9) |
|  |  |  |  |  |  | Mental function (thinking clearly and logically, memory) | 504 (75.9) | 123 (18.5) | 25 (3.8) | 10 (1.5) | 2 (0.3) |
|  |  |  |  |  |  | Vitality (e.g., healthy and energetic, weary, tired or feeble, exhausted) | 240 (36.1) | 241 (36.3) | 115 (17.3) | 56 (8.4) | 12 (1.8) |
|  |  |  |  |  |  | Sexual activities | 373 (56.2) | 130 (19.6) | 89 (13.4) | 26 (3.9) | 46 (6.9) |

Percentages may not total 100 by rows due to rounding.

Supplementary material 6 Distribution of EQ-5D-5L and 15D index values among respondents with physical conditions (N=1195)


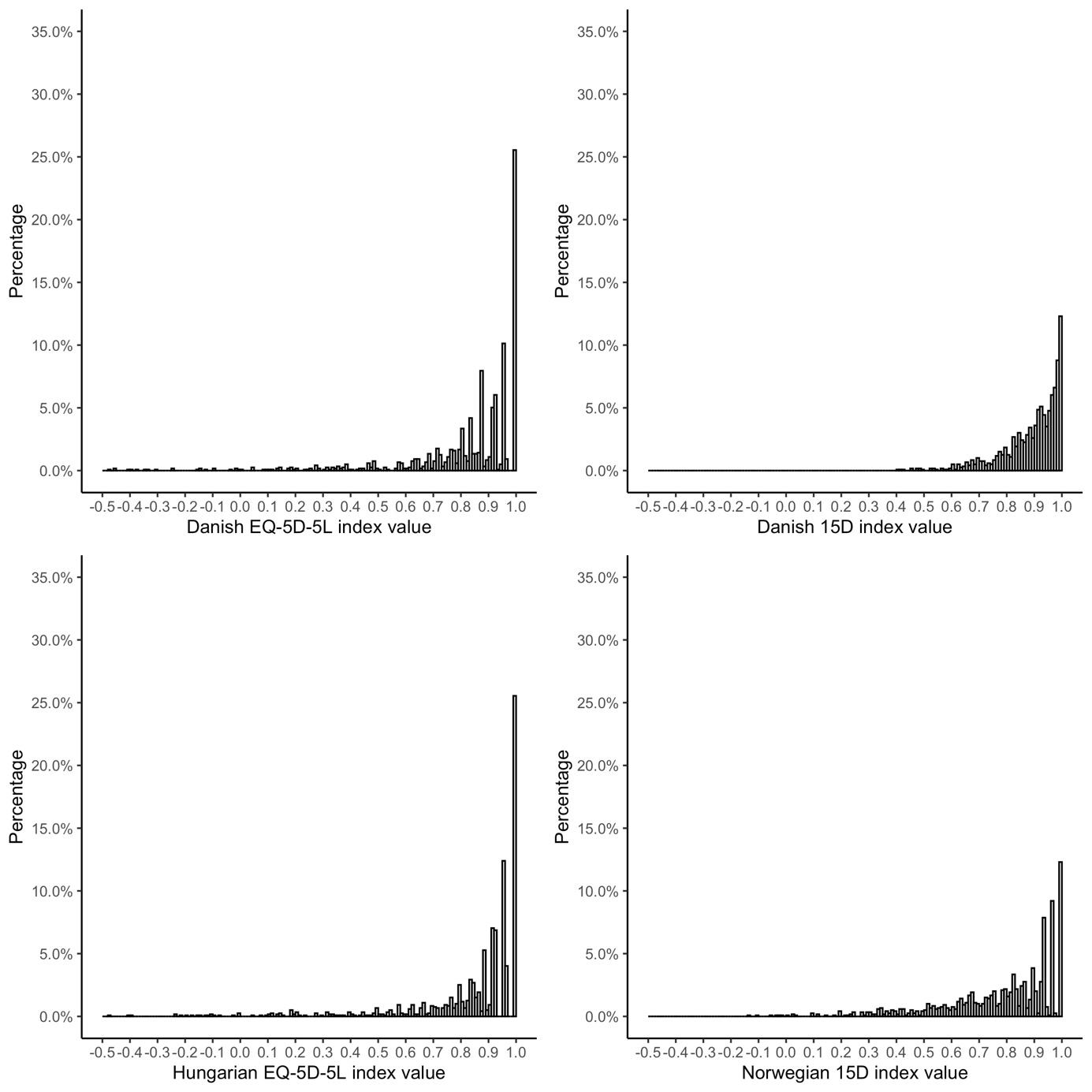


Supplementary material 7 Bland-Altman plot of EQ-5D-5L and 15D index values among respondents with physical conditions (N=1195)


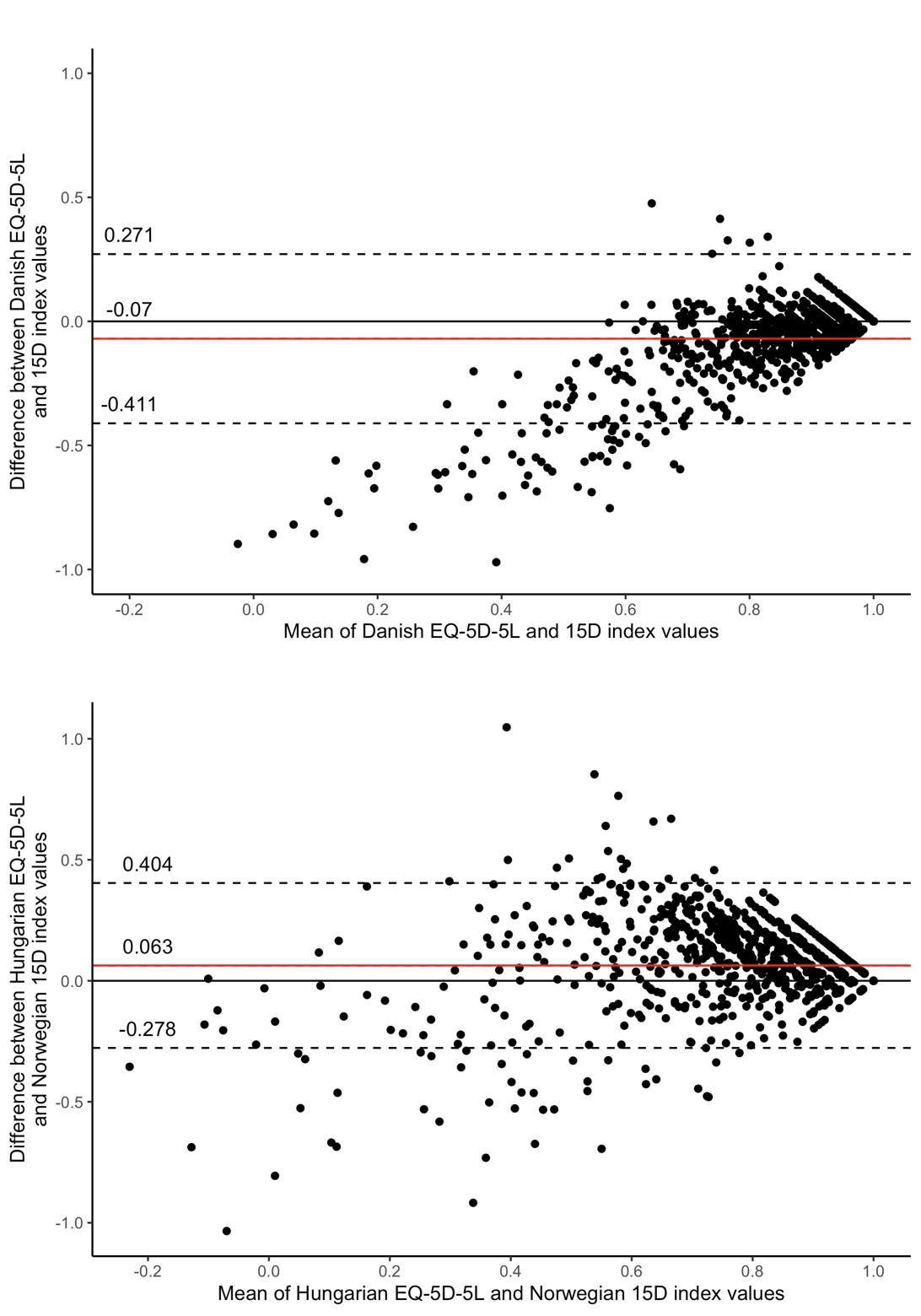


The horizontal red line represents the mean of the differences (D) between EQ-5D-5L and 15D index values, while the 95% confidence interval is represented by the dashed lines, which was obtained as D ± 1.96*SD (SD: standard deviation of the differences).

Supplementary material 8 Distribution of EQ-5D-5L and 15D index values among respondents with mental conditions (N=664)


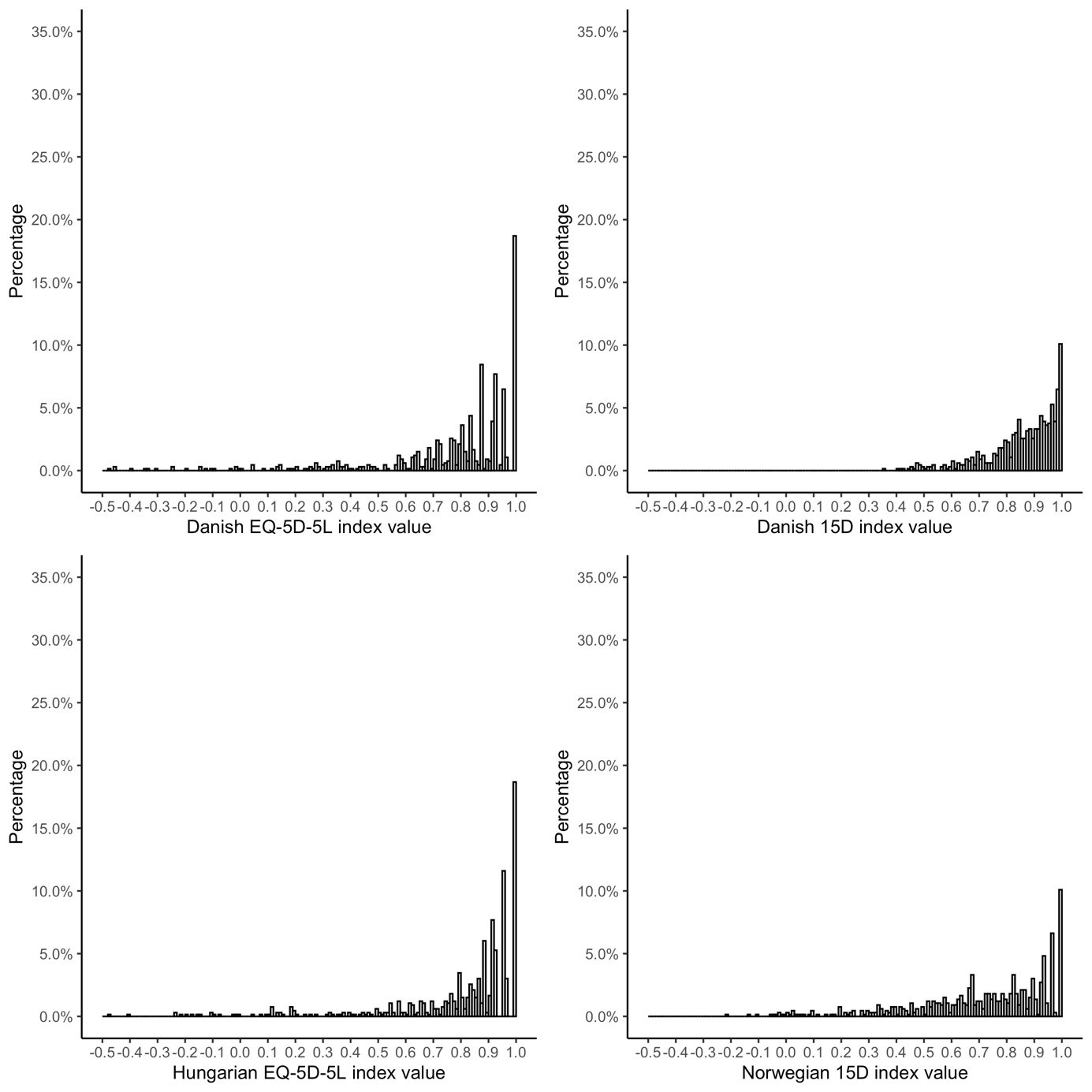


Supplementary material 9 Bland-Altman plot of EQ-5D-5L and 15D index values among respondents with mental conditions (N=664)


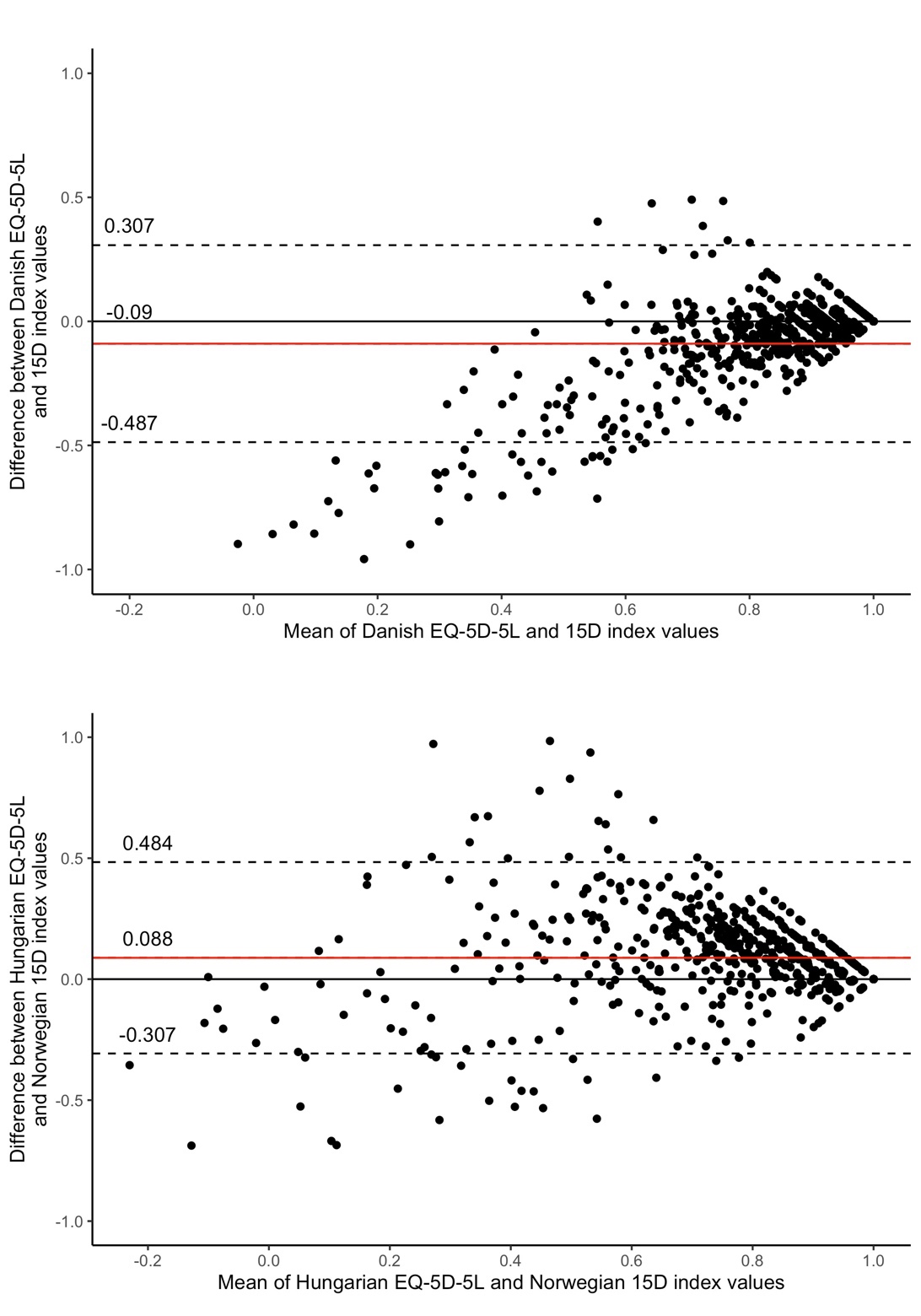


The horizontal red line represents the mean of the differences (D) between EQ-5D-5L and 15D index values, while the 95% confidence interval is represented by the dashed lines, which was obtained as D ± 1.96*SD (SD: standard deviation of the differences).

Supplementary material 10 Cross-tabulation of EQ-5D-5L and 15D responses between the corresponding dimensions among respondents with physical conditions (N=1195)

| EQ-5D-5L | 15D | | | | | Inconsistent response pairs, n (%) | Average size of inconsistencies |
| --- | --- | --- | --- | --- | --- | --- | --- |
| Dimensions | Level 1 | Level 2 | Level 3 | Level 4 | Level 5 |  |  |
| Mobility, n (%) | | | | | | | |
| Level 1 | 634 (94.6) | 25 (3.7) | 8 (1.2) | 0 (0.0) | 3 (0.4) | 95 (7.95) | 1.16 |
| Level 2 | 183 (65.6) | 86 (30.8) | 9 (3.2) | 0 (0.0) | 1 (0.4) |  |  |
| Level 3 | 52 (31.9) | 91 (55.8) | 16 (9.8) | 4 (2.5) | 0 (0.0) |  |  |
| Level 4 | 8 (10.3) | 22 (28.2) | 46 (59.0) | 2 (2.6) | 0 (0.0) |  |  |
| Level 5 | 0 (0.0) | 0 (0.0) | 1 (20.0) | 2 (40.0) | 2 (40.0) |  |  |
| Usual activities, n (%) | | | | | | | |
| Level 1 | 730 (91.5) | 58 (7.3) | 7 (0.9) | 3 (0.4) | 0 (0.0) | 52 (4.35) | 1.19 |
| Level 2 | 97 (41.5) | 122 (52.1) | 14 (6.0) | 1 (0.4) | 0 (0.0) |  |  |
| Level 3 | 23 (20.5) | 57 (50.9) | 24 (21.4) | 8 (7.1) | 0 (0.0) |  |  |
| Level 4 | 7 (14.3) | 11 (22.4) | 16 (32.7) | 14 (28.6) | 1 (2.0) |  |  |
| Level 5 | 0 (0.0) | 0 (0.0) | 0 (0.0) | 2 (100.0) | 0 (0.0) |  |  |
| Pain/discomfort (EQ-5D-5L) *and* Discomfort and symptoms (15D), n (%) | | | | | | | |
| Level 1 | 432 (91.1) | 36 (7.6) | 4 (0.8) | 2 (0.4) | 0 (0.0) | 85 (7.11) | 1.09 |
| Level 2 | 227 (52.1) | 193 (44.3) | 14 (3.2) | 2 (0.5) | 0 (0.0) |  |  |
| Level 3 | 6 (24.2) | 15 (44.8) | 16 (26.0) | 17 (4.5) | 1 (0.4) |  |  |
| Level 4 | 6 (10.9) | 15 (27.3) | 16 (29.1) | 17 (30.9) | 1 (1.8) |  |  |
| Level 5 | 0 (0.0) | 0 (0.0) | 1 (14.3) | 6 (85.7) | 0 (0.0) |  |  |
| Anxiety/depression (EQ-5D-5L) *and* Depression (15D), n (%) | | | | | | | |
| Level 1 | 626 (92.7) | 37 (5.5) | 10 (1.5) | 1 (0.1) | 1 (0.1) | 46 (3.85) | 1.22 |
| Level 2 | 130 (42.1) | 162 (52.4) | 15 (4.9) | 2 (0.6) | 0 (0.0) |  |  |
| Level 3 | 16 (10.7) | 66 (44.3) | 54 (36.2) | 11 (7.4) | 2 (1.3) |  |  |
| Level 4 | 4 (8.7) | 7 (15.2) | 19 (41.3) | 13 (28.3) | 3 (6.5) |  |  |
| Level 5 | 1 (6.2) | 1 (6.2) | 1 (6.2) | 7 (43.8) | 6 (37.5) |  |  |
| Anxiety/depression (EQ-5D-5L) *and* Distress (15D), n (%) | | | | | | | |
| Level 1 | 525 (77.8) | 135 (20.0) | 13 (1.9) | 2 (0.3) | 0 (0.0) | 55 (4.60) | 1.18 |
| Level 2 | 64 (20.7) | 201 (65.0) | 33 (10.7) | 9 (2.9) | 2 (0.6) |  |  |
| Level 3 | 13 (8.7) | 54 (36.2) | 52 (34.9) | 27 (18.1) | 3 (2.0) |  |  |
| Level 4 | 5 (10.9) | 6 (13.0) | 15 (32.6) | 13 (28.3) | 7 (15.2) |  |  |
| Level 5 | 0 (0.0) | 1 (6.2) | 1 (6.2) | 7 (43.8) | 7 (43.8) |  |  |

Percentages may not total 100 by rows due to rounding.

Supplementary material 11 Cross-tabulation of EQ-5D-5L and 15D responses between the corresponding dimensions among respondents with mental conditions (N=664)

| EQ-5D-5L | 15D | | | | | Inconsistent response pairs, n (%) | Average size of inconsistencies |
| --- | --- | --- | --- | --- | --- | --- | --- |
| Dimensions | Level 1 | Level 2 | Level 3 | Level 4 | Level 5 |  |  |
| Mobility, n (%) | | | | | | | |
| Level 1 | 325 (90.5) | 25 (7.0) | 8 (2.2) | 0 (0.0) | 1 (0.3) | 66 (9.94) | 1.09 |
| Level 2 | 99 (65.1) | 45 (29.6) | 7 (4.6) | 1 (0.7) | 0 (0.0) |  |  |
| Level 3 | 39 (36.4) | 55 (51.4) | 9 (8.4) | 4 (3.7) | 0 (0.0) |  |  |
| Level 4 | 4 (9.3) | 12 (27.9) | 26 (60.5) | 1 (2.3) | 0 (0.0) |  |  |
| Level 5 | 0 (0.0) | 0 (0.0) | 1 (33.3) | 1 (33.3) | 1 (33.3) |  |  |
| Usual activities, n (%) | | | | | | | |
| Level 1 | 363 (87.5) | 36 (8.7) | 12 (2.9) | 4 (1.0) | 0 (0.0) | 40 (6.02) | 1.20 |
| Level 2 | 56 (39.4) | 74 (52.1) | 10 (7.0) | 2 (1.4) | 0 (0.0) |  |  |
| Level 3 | 13 (18.1) | 33 (45.8) | 18 (25.0) | 8 (11.1) | 0 (0.0) |  |  |
| Level 4 | 4 (12.1) | 5 (15.2) | 12 (36.4) | 11 (33.3) | 1 (3.0) |  |  |
| Level 5 | 0 (0.0) | 0 (0.0) | 0 (0.0) | 2 (100.0) | 0 (0.0) |  |  |
| Pain/discomfort (EQ-5D-5L) *and* Discomfort and symptoms (15D), n (%) | | | | | | | |
| Level 1 | 203 (89.8) | 19 (8.4) | 4 (1.8) | 0 (0.0) | 0 (0.0) | 56 (8.43) | 1.10 |
| Level 2 | 113 (46.7) | 110 (45.5) | 16 (6.6) | 2 (0.8) | 1 (0.4) |  |  |
| Level 3 | 35 (24.1) | 64 (44.1) | 36 (24.8) | 9 (6.2) | 1 (0.7) |  |  |
| Level 4 | 4 (9.3) | 8 (18.6) | 13 (30.2) | 17 (39.5) | 1 (2.3) |  |  |
| Level 5 | 0 (0.0) | 0 (0.0) | 1 (12.5) | 6 (75.0) | 1 (12.5) |  |  |
| Anxiety/depression (EQ-5D-5L) *and* Depression (15D), n (%) | | | | | | | |
| Level 1 | 245 (90.1) | 18 (6.6) | 6 (2.2) | 3 (1.1) | 0 (0.0) | 40 (6.02) | 1.18 |
| Level 2 | 82 (40.0) | 106 (51.7) | 12 (5.9) | 4 (2.0) | 1 (0.5) |  |  |
| Level 3 | 14 (10.4) | 61 (45.5) | 48 (35.8) | 10 (7.5) | 1 (0.7) |  |  |
| Level 4 | 1 (2.7) | 6 (16.2) | 16 (43.2) | 12 (32.4) | 2 (5.4) |  |  |
| Level 5 | 1 (6.2) | 0 (0.0) | 3 (18.8) | 6 (37.5) | 6 (37.5) |  |  |
| Anxiety/depression (EQ-5D-5L) *and* Distress (15D), n (%) | | | | | | | |
| Level 1 | 209 (76.8) | 50 (18.4) | 8 (2.9) | 5 (1.8) | 0 (0.0) | 49 (7.38) | 1.20 |
| Level 2 | 38 (18.5) | 129 (62.9) | 26 (12.7) | 9 (4.4) | 3 (1.5) |  |  |
| Level 3 | 14 (10.4) | 45 (33.6) | 50 (37.3) | 23 (17.2) | 2 (1.5) |  |  |
| Level 4 | 1 (2.7) | 4 (10.8) | 15 (40.5) | 12 (32.4) | 5 (13.5) |  |  |
| Level 5 | 0 (0.0) | 1 (6.2) | 2 (12.5) | 5 (31.2) | 8 (50.0) |  |  |

Percentages may not total 100 by rows due to rounding.

Supplementary material 12 Correlation coefficients between 15D and EQ-5D-5L items among respondents with physical conditions (N=1195)

|  | **EQ-5D-5L** | | | | | **EQ VAS** | **EQ-5D-5L index value (Danish)** | **15D index value (Danish)** | **EQ-5D-5L index value (Hungarian)** | **15D index value (Norwegian)** |
| --- | --- | --- | --- | --- | --- | --- | --- | --- | --- | --- |
|  | **Mobility** | **Self-care** | **Usual activities** | **Pain/discomfort** | **Anxiety/depression** |  |  |  |  |  |
| **15D** | | | | | | | | | | |
| Mobility | **0.620** | 0.465 | 0.569 | 0.426 | 0.192 | -0.425 | -0.493 | -0.548 | -0.543 | -0.532 |
| Vision | 0.257 | 0.197 | 0.259 | 0.282 | 0.248 | -0.287 | -0.321 | -0.470 | -0.316 | -0.474 |
| Hearing | 0.204 | 0.231 | 0.197 | 0.190 | 0.123 | -0.214 | -0.217 | -0.359 | -0.227 | -0.354 |
| Breathing | 0.378 | 0.264 | 0.387 | 0.341 | 0.265 | -0.345 | -0.396 | -0.609 | -0.405 | -0.593 |
| Sleeping | 0.255 | 0.188 | 0.296 | 0.471 | 0.454 | -0.349 | -0.498 | -0.628 | -0.470 | -0.637 |
| Eating | 0.091 | 0.212 | 0.135 | 0.111 | 0.111 | -0.129 | -0.147 | -0.267 | -0.142 | -0.263 |
| Speech | 0.128 | 0.204 | 0.175 | 0.179 | 0.246 | -0.185 | -0.235 | -0.371 | -0.217 | -0.366 |
| Excretion | 0.241 | 0.177 | 0.252 | 0.327 | 0.247 | -0.273 | -0.339 | -0.518 | -0.331 | -0.531 |
| Usual activities | 0.508 | 0.475 | **0.653** | 0.509 | 0.355 | -0.499 | -0.585 | -0.664 | -0.602 | -0.658 |
| Mental function | 0.218 | 0.232 | 0.262 | 0.321 | 0.352 | -0.240 | -0.366 | -0.509 | -0.347 | -0.499 |
| Discomfort and symptoms | 0.405 | 0.282 | 0.441 | **0.601** | 0.472 | -0.468 | -0.609 | -0.724 | -0.589 | -0.727 |
| Depression | 0.188 | 0.193 | 0.284 | 0.416 | **0.717** | -0.376 | -0.576 | -0.679 | -0.507 | -0.689 |
| Distress | 0.192 | 0.142 | 0.277 | 0.421 | **0.678** | -0.349 | -0.555 | -0.651 | -0.490 | -0.676 |
| Vitality | 0.402 | 0.280 | 0.476 | 0.555 | 0.507 | -0.537 | -0.627 | -0.789 | -0.605 | -0.792 |
| Sexual activities | 0.354 | 0.246 | 0.419 | 0.424 | 0.319 | -0.392 | -0.455 | -0.632 | -0.455 | -0.638 |
| **EQ VAS** | -0.482 | -0.362 | -0.483 | -0.584 | -0.397 | - | - | - | - | - |
| **EQ-5D-5L index value (Danish)** | -0.664 | -0.483 | -0.680 | -0.835 | -0.736 | 0.608 | - | - | - | - |
| **15D index value (Danish)** | -0.506 | -0.380 | -0.550 | -0.652 | -0.591 | 0.594 | 0.736 | - | - | - |
| **EQ-5D-5L index value (Hungarian)** | -0.728 | -0.529 | -0.727 | -0.845 | -0.630 | 0.611 | 0.962 | 0.694 | - | - |
| **15D index value (Norwegian)** | -0.498 | -0.369 | -0.542 | -0.652 | -0.601 | 0.594 | 0.727 | 0.998 | 0.683 | - |

Pearson correlation coefficient was calculated for the continuous index values, while Spearman’s rank correlation for the ordinal dimensions.

p < 0.05 for all correlation coefficients (two-tailed).

Corresponding dimensions between EQ-5D-5L and 15D are in bold.

Supplementary material 13 Correlation coefficients between 15D and EQ-5D-5L items among respondents with mental conditions (N=664)

|  | **EQ-5D-5L** | | | | | **EQ VAS** | **EQ-5D-5L index value (Danish)** | **15D index value (Danish)** | **EQ-5D-5L index value (Hungarian)** | **15D index value (Norwegian)** |
| --- | --- | --- | --- | --- | --- | --- | --- | --- | --- | --- |
|  | **Mobility** | **Self-care** | **Usual activities** | **Pain/discomfort** | **Anxiety/depression** |  |  |  |  |  |
| **15D** | | | | | | | | | | |
| Mobility | **0.559** | 0.473 | 0.543 | 0.409 | 0.195 | -0.399 | -0.448 | -0.572 | -0.507 | -0.560 |
| Vision | 0.299 | 0.307 | 0.325 | 0.319 | 0.292 | -0.284 | -0.382 | -0.538 | -0.387 | -0.543 |
| Hearing | 0.188 | 0.284 | 0.220 | 0.197 | 0.177 | -0.216 | -0.232 | -0.412 | -0.248 | -0.410 |
| Breathing | 0.415 | 0.367 | 0.436 | 0.409 | 0.320 | -0.357 | -0.456 | -0.672 | -0.481 | -0.661 |
| Sleeping | 0.255 | 0.228 | 0.327 | 0.455 | 0.457 | -0.336 | -0.501 | -0.640 | -0.482 | -0.644 |
| Eating | 0.067* | 0.297 | 0.162 | 0.127 | 0.161 | -0.088 | -0.181 | -0.379 | -0.180 | -0.375 |
| Speech | 0.111 | 0.323 | 0.234 | 0.195 | 0.282 | -0.154 | -0.281 | -0.461 | -0.271 | -0.456 |
| Excretion | 0.224 | 0.227 | 0.284 | 0.311 | 0.289 | -0.237 | -0.343 | -0.571 | -0.344 | -0.581 |
| Usual activities | 0.466 | 0.446 | **0.625** | 0.515 | 0.391 | -0.474 | -0.582 | -0.708 | -0.600 | -0.705 |
| Mental function | 0.207 | 0.339 | 0.294 | 0.306 | 0.395 | -0.228 | -0.411 | -0.578 | -0.400 | -0.569 |
| Discomfort and symptoms | 0.407 | 0.342 | 0.458 | **0.616** | 0.501 | -0.503 | -0.624 | -0.754 | -0.613 | -0.756 |
| Depression | 0.191 | 0.266 | 0.325 | 0.418 | **0.715** | -0.318 | -0.608 | -0.723 | -0.544 | -0.732 |
| Distress | 0.180 | 0.186 | 0.320 | 0.445 | **0.686** | -0.333 | -0.583 | -0.690 | -0.520 | -0.705 |
| Vitality | 0.408 | 0.319 | 0.476 | 0.535 | 0.529 | -0.500 | -0.618 | -0.784 | -0.607 | -0.784 |
| Sexual activities | 0.346 | 0.286 | 0.403 | 0.395 | 0.369 | -0.350 | -0.461 | -0.652 | -0.463 | -0.664 |
| **EQ VAS** | -0.516 | -0.343 | -0.507 | -0.606 | -0.413 | - | - | - | - | - |
| **EQ-5D-5L index value (Danish)** | -0.641 | -0.516 | -0.683 | -0.821 | -0.800 | 0.590 | - | - | - | - |
| **15D index value (Danish)** | -0.478 | -0.440 | -0.567 | -0.634 | -0.630 | 0.505 | 0.702 | - | - | - |
| **EQ-5D-5L index value (Hungarian)** | -0.717 | -0.569 | -0.736 | -0.839 | -0.696 | 0.599 | 0.969 | 0.679 | - | - |
| **15D index value (Norwegian)** | -0.472 | -0.431 | -0.561 | -0.633 | -0.637 | 0.507 | 0.697 | 0.998 | 0.671 | - |

Pearson correlation coefficient was calculated for the continuous index values, while Spearman’s rank correlation for the ordinal dimensions.

p < 0.05 for all correlation coefficients (two-tailed), except for those marked with asterisks.

Corresponding dimensions between EQ-5D-5L and 15D are in bold.

# References

1. Hungarian Central Statistical Office. Microcensus 2016. <https://www.ksh.hu/docs/eng/xftp/idoszaki/microcensus2016/microcensus_2016_3.pdf>. Accessed December 1, 2021.

2. Hungarian Central Statistical Office. Tehetünk az egészségünkért – ELEF2019 gyorsjelentés. <https://www.ksh.hu/docs/hun/xftp/idoszaki/elef/te_2019/index.html>. Accessed March 11, 2022.
